# Structural classification, biosynthesis, metabolic engineering and ecological functions for the terpenes produced in tobacco

**DOI:** 10.3389/fpls.2026.1838605

**Published:** 2026-06-16

**Authors:** Xiujuan Xu, Xinyu Li, Damiao Liu, Jun Hu, Baoan Deng, Chunqiang Yang, Yuan He, Weichen Zhang, Xiangyu Liu, Weiguo Li, Jianjun Qiao, Shan Liu, Xiaobin Li, Shengli Wang

**Affiliations:** 1 Zhengzhou Tobacco Research Institute of National Tobacco Corporation, Zhengzhou, China; 2 Zhejiang Institute of Tianjin University (Shaoxing), Shaoxing, China; 3 China tobacco Shaanxi industrial Co., Ltd., Xi’an, China; 4 State Key Laboratory of Synthetic Biology, Tianjin University, Tianjin, China; 5 School of Chemical Engineering and Technology, Tianjin University, Tianjin, China

**Keywords:** aroma, bioactivities, biosynthesis, terpenes, tobacco

## Abstract

Tobacco is a globally economic industrial crop due to its distinctive flavor and important commercial value. Diseases, pests and insufficient aroma of tobacco are not only limited to affecting their quantity and quality but also affect their industrial availability. Terpenoid compounds are a large class of secondary metabolites present in tobacco (*Nicotiana* genus), which can protect tobacco against biotic stress and influence the flavor and fragrance of tobacco products. This is the first systematic review of the chemical structures, biosynthesis, metabolic engineering and ecological functions of terpenes in tobacco. A total of 300 terpenes reported in tobacco were summarised and classified according to their chemical structure characteristics. A brief overview of the biosynthesis, key genes and metabolic strategies for terpenes in tobacco was carried out. Finally, the functions of terpenes in tobacco aroma and enhancing tobacco resistance against insect and disease have also been discussed.

## 1 Introduction

*Nicotiana tabacum* L., also termed tobacco, is a crucial economic crop with huge commercial, medicinal and chemical value. Specially, tobacco has been used for a long time to produce nicotine-containing products such as cigarettes, cigars, and smokeless tobacco, which has led to the widespread use of tobacco. Studies have shown biotic stress (pests and diseases) and abiotic stress (drought, flood and high temperature) are one of the primary challenges to tobacco production safety, which affect the normal growth and development of tobacco and reduce the yield and quality of flue-cured tobacco (Fei et al., 2025; Zong et al., 2023). For example, tobacco mosaic virus (TMV) is regarded as plant cancer, which makes the tobacco show symptoms such as deformity of leaves, leaf curling and mottling, dwarfing of plants and stunted growth, seriously affecting the quality of tobacco leaves (Ellis et al., 2020). In addition, insufficient aroma of tobacco leaves is another major factor limiting the quality of tobacco leaves and their industrial availability. The chemical composition is the fundamental factor that determines the aroma of tobacco. Species within *Nicotiana* show remarkable chemical diversity and it was reported that more than 2500 kinds of components in *N. tabacum* have been identified, including terpenoid, polyphenols, flavonoids, organic acids and alkaloids (Gui et al., 2024; Liu et al., 2023a). Among them, terpenoid are key aroma compounds determining the different style features of tobacco products. Identifying the key aroma compounds that influence the style characteristics of tobacco leaves has been a hotspot in recent years.

Terpenoid compounds represent the largest group of secondary metabolites that are nearly ubiquitous in almost all classes of living organisms. Previous phytochemical investigations on tobacco showed that *N. tabacum* is rich in terpenoids, especially cembranoids, labdanes and carotenoid degradation products (Popova et al., 2019a; Xu et al., 2024a; Yan et al., 2019). In some cultivars, these terpenoids represent up to 10% of the leaf dry weight under favorable conditions (Wang and Wagner, 2003). Some of terpenoids have been shown to play an important role in tobacco self-defense against pest and pathogen attack. For instance, tabasesquiterpenes B and solanesol in tobacco have demonstrated the ability to counter TMV infection (Shang et al., 2016; Yan et al., 2015). Previous studies have demonstrated that cembratrien-diols exhibited excellent insecticidal activity *in vivo* and *in vitro* bioactivity studies (Mischko et al., 2018; Yan et al., 2019). In addition, some terpenoids, especially carotenoid degradation products and diterpenoid degradation products, are also significant aroma precursors in tobacco. Their degradation products, including *β*-ionone, geranylacetone, solanone and solanofuran, produce various compounds with distinct and pleasant aromas, closely related to the aroma characteristics and intensity of tobacco leaves (He et al., 2021). Although the large number of terpenes have been isolated and characterized from tobacco, their biosynthetic pathways and biological functions have not been fully studied.

Previous studies have extensively discussed the diterpenoids biosynthesis and bioactivities in tobacco (Wu et al., 2025; Xu et al., 2024a). However, the biosynthesis and ecological functions of all terpenes in tobacco have not been systematically discussed. In this review, we present a comprehensive overview of the chemical structures, biosynthesis and key synthase genes for terpenes in tobacco. We also discuss metabolic engineering approaches in tobacco terpenes metabolism, as well as ecological functions of terpenes in tobacco.

## 2 Methodology

To summarise the natural terpenes obtained from tobacco and study their chemical structures, biosynthesis and ecological functions, the search terms “tobacco terpenes”, “biosynthesis of tobacco terpenes” and “functions of tobacco terpenes” were used for data collection. This review includes suitable original articles obtained from databases such as Web of Science, PubMed, SciFinder, China National Knowledge Infrastructure, Google Scholar, and ScienceDirect from 1961-2025. In this work, we selected only terpenes that could be extracted from tobacco and excluded those that could be synthesised. A total of 300 terpenes from *N. tabacum* were summarized (**Table 1**) and 22 key functional genes associated with terpene biosynthesis in tobacco were collected (**Table 2**). ChemDraw 20.0 software was used to draw the chemical structures of terpenes.

**Table 1:** Terpenes found in tobacco.

NO	Compounds	Sources	References
1	Myrcene	VOCs	(Raguso et al., 2006)
2	Linalool	VOCs	(Berenguer et al., 2021)
3	Nerol	VOCs	(Couto et al., 2024)
4	(Z)-*β*-ocimene	VOCs	(Raguso et al., 2006)
5	(E)-*β*-ocimene	VOCs	(Raguso et al., 2006)
6	Geraniol	VOCs	(Couto et al., 2024)
7	(E)-Geranyl acetone	VOCs	(Popova et al., 2019c)
8	(E)-2,6-dimethyl-3,7-octadiene-2,6-diol	VOCs	(Raguso et al., 2006)
9	2,7-Dimethyl-1,6-octadiene	VOCs	(Berenguer et al., 2021)
10	Trans-2,6-Dimethyl-2–6-octadiene	VOCs	(Berenguer et al., 2021)
11	*α*-Terpineol	VOCs	(Raguso et al., 2006)
12	Limonene	VOCs	(Berenguer et al., 2021)
13	*α*-Terpinolene	VOCs	(Couto et al., 2024)
14	Thymol	VOCs	(Popova et al., 2019c)
15	Chrysanthemone	VOCs	(Couto et al., 2024)
16	2,6,6-trimethyl-2-cyclohex ene-1,4-dione (4-oxoisophorone)	VOCs	(Popova et al., 2019c)
17	2,6,6-trimethyl-1,4 cyclohexadione	VOCs	(Popova et al., 2019c)
18	1,3,3-trimethyl-7-oxa-bicy clo [4.1.0] heptan-2,5-dione	VOCs	(Popova et al., 2019c)
19	(Z)-Linalool oxide	VOCs	(Popova et al., 2019c)
20	p-Cymene	VOCs	(Berenguer et al., 2021)
21	Menthol	Additives	(Kopa and Pawliczak, 2020)
22	*α*-Pinene	VOCs	(Raguso et al., 2006)
23	*β*-Pinene	VOCs	(Berenguer et al., 2021)
24	Camphene	VOCs	(Couto et al., 2024)
25	Sabinene	VOCs	(Raguso et al., 2006)
26	*α*-Thujene	VOCs	(Couto et al., 2024)
27	1,8-Cineole	VOCs	(Raguso et al., 2006)
28	Camphor	VOCs	(Couto et al., 2024)
29	*α*-Farnesene	VOCs	(Raguso et al., 2006)
30	*β*-Farnesene	VOCs	(Raguso et al., 2006)
31	Farnesol	VOCs	(Couto et al., 2024)
32	Nerolidol	VOCs	(Raguso et al., 2006)
33	*α*-Humulene	VOCs	(Raguso et al., 2006)
34	Germacrene D	VOCs	(Raguso et al., 2006)
35	*α*-Zingiberene	VOCs	(Couto et al., 2024)
36	*β*-Sesquiphelladrene	VOCs	(Couto et al., 2024)
37	*β*-Caryophyllene	VOCs	(Raguso et al., 2006)
38	*α*-Muuroline	VOCs	(Couto et al., 2024)
39	Calamenene	VOCs	(Couto et al., 2024)
40	*β*-Selinene	VOCs	(Couto et al., 2024)
41	Acoradiene	VOCs	(Couto et al., 2024)
42	*α*-Cedrene	VOCs	(Raguso et al., 2006)
43	*β*-Cedrene	VOCs	(Couto et al., 2024)
44	E-*α*-bergamotene	VOCs	(Raguso et al., 2006)
45	Aromadendrene	VOCs	(Couto et al., 2024)
46	Caryophylleneoxide	VOCs	(Couto et al., 2024)
47	Sativene	VOCs	(Couto et al., 2024)
48-49	Nicosesquiterpene A-B	Leaves	(Shen et al., 2016a)
50	Glutinosone	Leaves	(Shen et al., 2016a)
51	Capsidiol	Leaves	(Shen et al., 2016a)
52	1-*β*-hydroxy-*α*-cyperone	Leaves	(Shen et al., 2016a)
53	Arundinol B	Leaves	(Shen et al., 2016a)
54-55	Nicotianasesterpenes A-B	Leaves	(Shen et al., 2016b)
56-61	Nicotabacoside A-F	Leaves	(Yang et al., 2014)
62	Nicotabin A	Leaves	(Feng et al., 2017)
63-65	Tabasesquiterpene A-C	Leaves	(Shang et al., 2016)
66	Balsamiferine B	Leaves	(Shang et al., 2016)
67	Samboginone	Leaves	(Shang et al., 2016)
68	Ent-4(15)-eudesmen-1*α*,11-diol	Leaves	(Shang et al., 2016)
69	Tabsesquiterpene A	Leaves	(Chen et al., 2014)
70	Nicotterpene A	Leaves	(Yang et al., 2013)
71	7-isopropyl-3,5-dimethoxy-1-methyl-naphthale n-2-ol	Leaves	(Shan et al., 2016)
72	7-isopropyl-2,5-dimethoxy-1-methyl-naphthalene	Leaves	(Shan et al., 2016)
73	(3-isopropyl-1,6-dimethoxy-naphthalen-5-yl) methanol	Leaves	(Shan et al., 2016)
74	10*β*-eudesm-4-en-3-one-11,12-diol-12-O-*β*-glucopyranoside	Flowers	(Yang et al., 2021)
75-76	Nicotiasesquiterpenes A-B	Flowers	(Xu et al., 2022)
77	methyl 4-isopropyl-7-methoxy-6-methylnaphthalene-1-carboxylate	Stems	(Yang et al., 2019)
78	methyl 2-hydroxy-4-isopropyl-7-methoxy-6-methylnaphthalene-1-carboxylate	Stems	(Yang et al., 2019)
79	methyl 2-hydroxy-6-(hydroxymethyl)-4-isopropyl-7-methoxynaphthalene-1-carboxylate	Stems	(Yang et al., 2019)
80	Tobterpene B	Stems	(Hu et al., 2015)
81-168	isopropyl cembranoids (1-88)	Flowers; Leaves	(Xu et al., 2024a)
169-183	seco-cembranoids (89–103)	Flowers; Leaves	(Xu et al., 2024a)
184-203	chain cembranoids (104–123)	Flowers; Leaves	(Xu et al., 2024a)
204-211	polycyclic cembranoids (124–131)	Flowers; Leaves	(Xu et al., 2024a)
212-230	epoxy side chain labdanes (132-150)	Flowers; Leaves	(Xu et al., 2024a)
231-251	epoxy-free side chain labdanes (151–171)	Flowers; Leaves	(Xu et al., 2024a)
252-267	Nicotabacins A−P	Leaves	(Yan et al., 2025)
268-276	Cigarcembrane A-I	Leaves	(Zhang et al., 2025)
277	Squalene	Leaves	(Popova et al., 2019c)
278	*α*-Tocopherol	Leaves	(Popova et al., 2019c)
279	*β*-Stigmasterol	Leaves	(Popova et al., 2019c)
280	Carnosic acid	Leaves	(Popova et al., 2020)
281	Betulin	Leaves	(Popova et al., 2020)
282	Betulinic acid	Leaves	(Popova et al., 2020)
283	Oleanolic acid	Leaves	(Popova et al., 2020)
284	Ursolic acid	Leaves	(Popova et al., 2020)
285	Sterol	Leaves	(Shang et al., 2019)
286	*β*-Carotene	Leaves	(Wahlberg and Enzell, 1987)
287	Lutein	Leaves	(Wahlberg and Enzell, 1987)
288	Neoxanthin	Leaves	(Wahlberg and Enzell, 1987)
289	Violaxthin	Leaves	(Wahlberg and Enzell, 1987)
290	Phytofluene	Leaves	(Wahlberg and Enzell, 1987)
291	Flavoxanthin	Leaves	(Wahlberg and Enzell, 1987)
292	Antheraxanthin	Leaves	(Wahlberg and Enzell, 1987)
293	Apocarotenal	Flowers	(Xiao et al., 2022)
294	Zeaxanthin	Flowers	(Xiao et al., 2022)
295	Echinenone	Flowers	(Xiao et al., 2022)
296	*α*-Carotene	Flowers	(Xiao et al., 2022)
297	*β*-Cryptoxanthin	Flowers	(Xiao et al., 2022)
298	Lycopene	Flowers	(Xiao et al., 2022)
299	Phytoene	Flowers	(Xiao et al., 2022)

**Table 2:** Summary of identified key genes associated with terpene biosynthesis in tobacco.

Gene name	Species	Substrate	Products	References
NtTPS2	*Nicotiana tabacum*	GPP	geraniol, nerol	(Liu et al., 2021)
NtTPS25	*Nicotiana tabacum*	GPP	*E*-*β*-Ocimene	(Li, 2021)
CIN	*Nicotiana noctiflora*	GPP	*α*-pinene, *β*-pinene, sabinene, *β*-myrcene, limonene, 1,8-cineole	(Fähnrich et al., 2014, 2012)
EAS	*Nicotiana tabacum*	FPP	5-epi-aristolochene	(Starks et al., 1997)
NtTPS7	*Nicotiana tabacum*	FPP	*β*-caryophyllene	(Cheng et al., 2022)
NtTPS21	*Nicotiana tabacum*	FPP	*β*-caryophyllene	(Yang et al., 2022)
NtTPS126	*Nicotiana tabacum*	FPP	*β*-longipinene, *α*-eudesmol	(Yang et al., 2024a)
NaTPS38	*Nicotiana attenuata*	FPP	(E)-*α*-bergamotene	(Zhou et al., 2017)
NaGLS	*Nicotiana attenuata*	GGPP	geranyllinalool; 16-OH-geranyllinalool	(Forestier et al., 2021)
NtCPS2	*Nicotiana tabacum*	GGPP	8-hydroxy-copalyl diphosphate	(Sallaud et al., 2012)
NtCBTS	*Nicotiana tabacum*	GGPP	cembratrien-ol	(Ennajdaoui et al., 2010)
NtABS	*Nicotiana tabacum*	8-hydroxy-copalyl diphosphate	Z-abienol	(Sallaud et al., 2012)
NtSPS1	*Nicotiana tabacum*	IPP/DMAPP	solanesyl diphosphate	(Yan et al., 2017)
NaOSC1	*Nicotiana attenuata*	2,3-oxidosqualene	lupeol, dammarenediol II, 3-alpha,20-lupanediol, and 7 other triterpene scaffolds	(Yang et al., 2023)
NaOSC2	*Nicotiana attenuata*		*β*-amyrin	
CYP71D20	*Nicotiana tabacum*	5-epi-aristolochene	capsidiol	(Ralston et al., 2001)
CYP71D16	*Nicotiana attenuata*	*α*-cembratrien-ol	*α*-cembratrien-diol	(Wang et al., 2001)
		*β*-cembratrien-ol	*β*-cembratrien-diol	
CYP716A419	*Nicotiana attenuata*	*β*-amyrin	erythrodiol; oleanolic acid	(Yang et al., 2024b)
		lupeol	betulin, betulinic acid	
		lupanediol	28-hydroxy lupanediol	
CYP716C87	*Nicotiana attenuata*	*β*-amyrin	2*α*-hydroxyl *β*-amyrin	
		lupeol	2*α*-hydroxy lupeol	
		lupanediol	2*α*-hydroxy lupanediol	
CYP716E107	*Nicotiana attenuata*	oleanolic acid	6*β*-hydroxy oleanolic acid	
NtCCD1/4/7	*Nicotiana tabacum*	*β*-carotene	*β*-ionone	(Liu et al., 2026)
NtCCD10	*Nicotiana tabacum*	phytoene	geranylacetone	(Li et al., 2022)
		*β*-carotene	*β*-ionone	

## 3 Structural classification

According to the number of isoprene units, terpenes can be grouped into monoterpenes (C_10_H_16_), sesquiterpenes (C_15_H_24_), diterpenes (C_20_H_32_), triterpenes (C_30_H_48_), tetraterpenes (C_40_H_64_) and polyterpenes (C_5_H_8_)n (Wang et al., 2021). More than 80,000 terpenes have been identified to date in nature (Christianson, 2017), and there are 300 terpenes with different structures found in volatile organic compounds (VOCs), leaf and flower of tobacco (**Table 1**).

### 3.1 Monoterpenes

**Figure 1:**
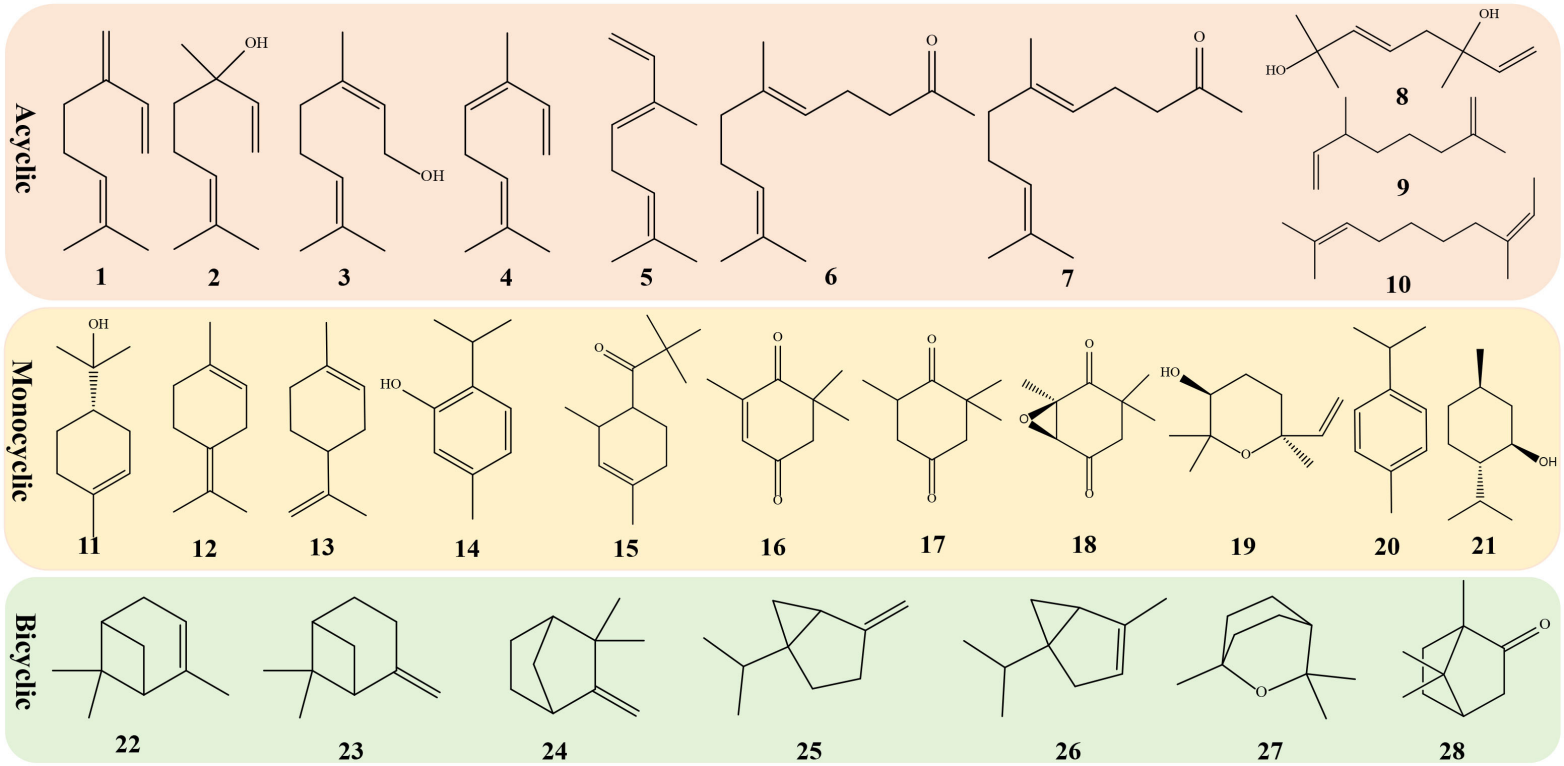
Chemical structures of monoterpenes (1−28) found from *Nicotiana*.

Monoterpenes are a class of terpenes that consist of two isoprene units. Depending on their structure, monoterpenes are classified as acyclic, monocyclic, or bicyclic compounds. These compounds were divided to two major chemical groups including hydrocarbons and oxygenated monoterpenes (acetate, alcohols, ketones and aldehydes). It has been reported that monoterpene hydrocarbons and oxygenated monoterpenes are the most ubiquitous compounds in floral volatile organic compounds (VOCs) emitted by *Nicotiana* species (**Table 1** and **Figure 1**) (Couto et al., 2024; Fähnrich et al., 2011; Raguso et al., 2006). The scent bouquets of flowers of *Nicotiana* species, particularly those of section *Alatae*, can produce and emit characteristic floral monoterpene volatiles including myrcene, *α*-terpineol, limonene, *α*-pinene, *β*-pinene, sabinene and 1,8-cineole. Since 1,8-cineole was the major compound, this set of monoterpenes was called ‘cineole cassette’ (Fähnrich et al., 2011). Particularly, menthol, a cyclic monoterpene alcohol with well-known cooling characteristics, has been widely added into commercial tobacco products such as e-cigarettes, cigars and hookah tobacco.

### 3.2 Sesquiterpenes

**Figure 2:**
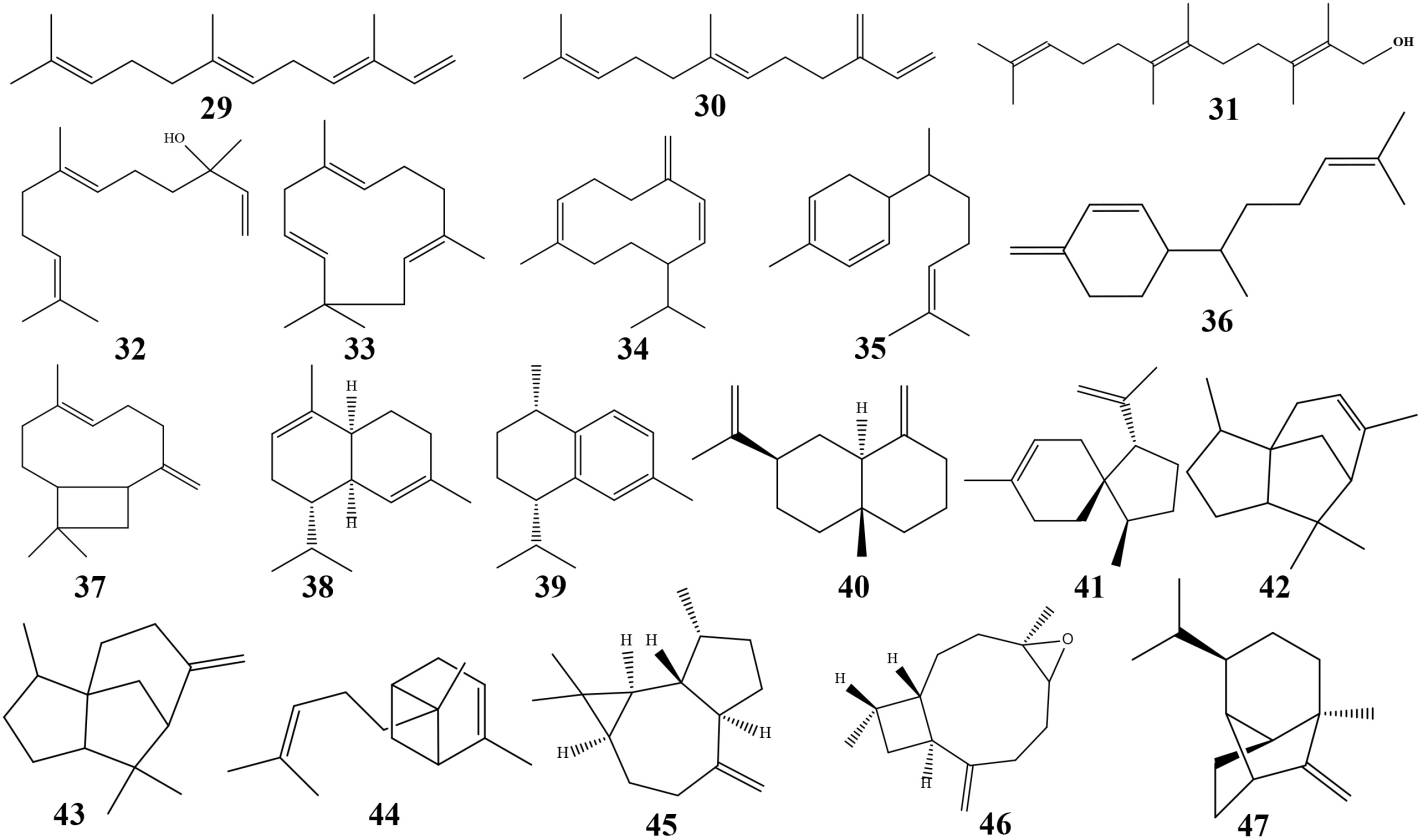
Chemical structures of sesquiterpenes (29−47) found in VOCs of *Nicotiana*.

**Figure 3:**
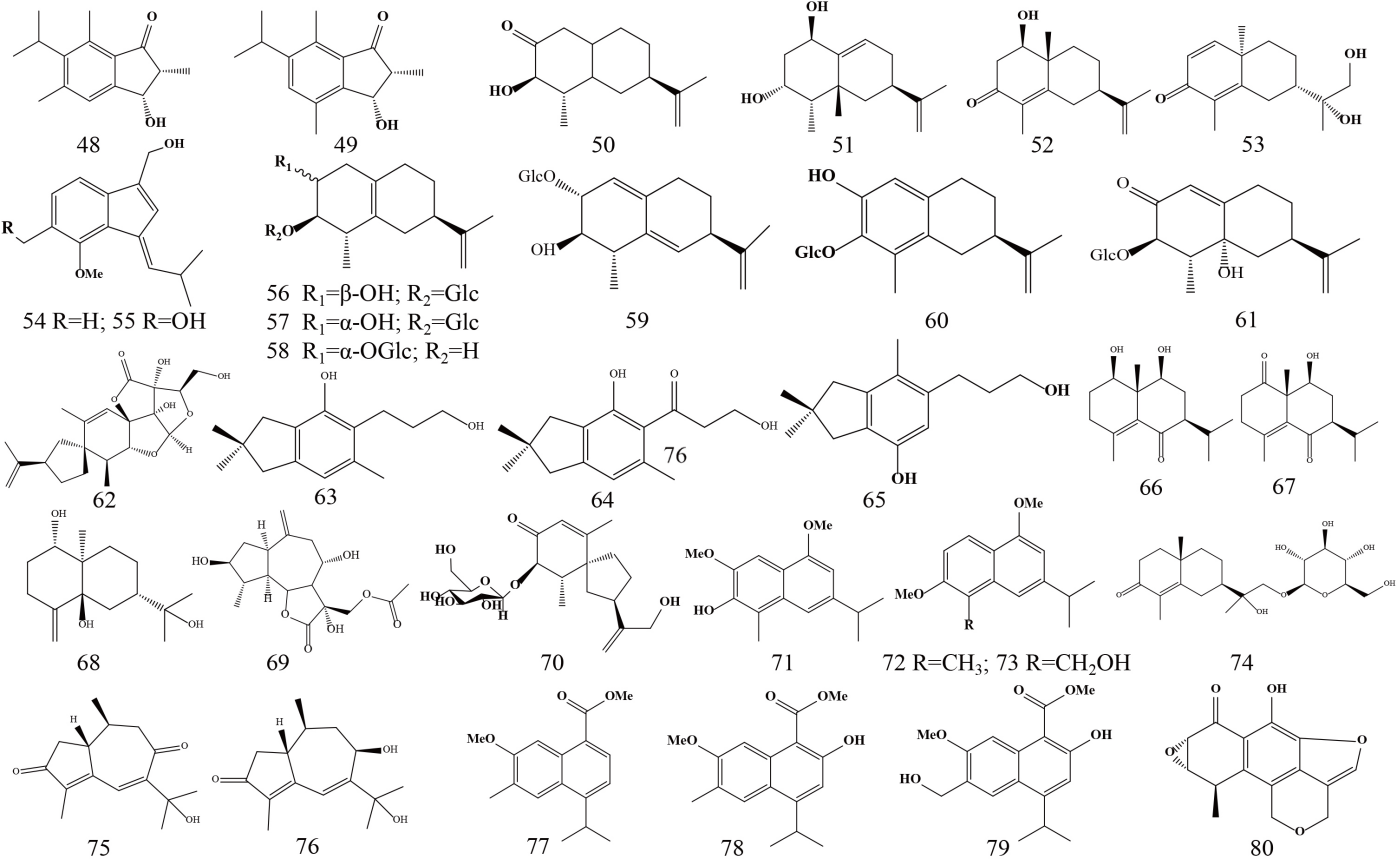
Chemical structures of sesquiterpenoid derivatives (48−80) isolated from *Nicotiana*.

Sesquiterpenes form a structurally diverse family of natural products consisting of three isoprene building units. Based on their carbon ring number, sesquiterpene frameworks can be classified into acyclic, monocyclic, bicyclic, tricyclic, and multicyclic. They occur in nature as hydrocarbons or in oxygenated forms including lactones, alcohols, acids, aldehydes, and ketones. To date, more and more sesquiterpenoids have been found and characterized from different sources of tobacco (Gui et al., 2024). Previous studies found there were 19 sesquiterpene hydrocarbons and alcohols in the identified VOCs of *Nicotiana* species, including acyclic sesquiterpenes (*α*-farnesene, *β*-farnesene, farnesol and nerolidol), monocyclic sesquiterpenes (*α*-humulene, germacrene D, *α*-zingiberene and *β*-sesquiphelladrene), bicyclic sesquiterpenes (*β*-caryophyllene, *α*-muuroline, calamenene, *β*-selinene and acoradiene) and others (**Table 1**; **Figure 2**) (Couto et al., 2024; Raguso et al., 2006). Besides, many sesquiterpenoid derivatives possessing diversified skeletons have been isolated and their structures have been isolated from leaves, flowers and stems of *Nicotiana* (**Table 1**; **Figure 3**) (Gui et al., 2024). Shen et al. isolated two new pterosin-type sesquiterpenes bearing an isopropyl moiety (nicosesquiterpene A and B) (Shen et al., 2016a) and two unreported sesquiterpenoids (nicotianasesterpenes A and B) (Shen et al., 2016b) from the leaves of *N. tabacum*. Six unreported 14-noreudesmane sesquiterpenoid glycosides, nicotabacosides A−F, have been discovered in 90% EtOH extracts from leaves (Yang et al., 2014). Xu et al. obtained two new guaiane -type sesquiterpenes, nicotiasesquiterpenes A and B, from the flowers of *N. tabacum* (Xu et al., 2022). In addition, three new sesquiterpenes 77–78 an undescribed eremophilane sesquiterpenoid (tobterpene B) were also isolated from *N. tabacum* stems.

### 3.3 Diterpenes

**Figure 4:**
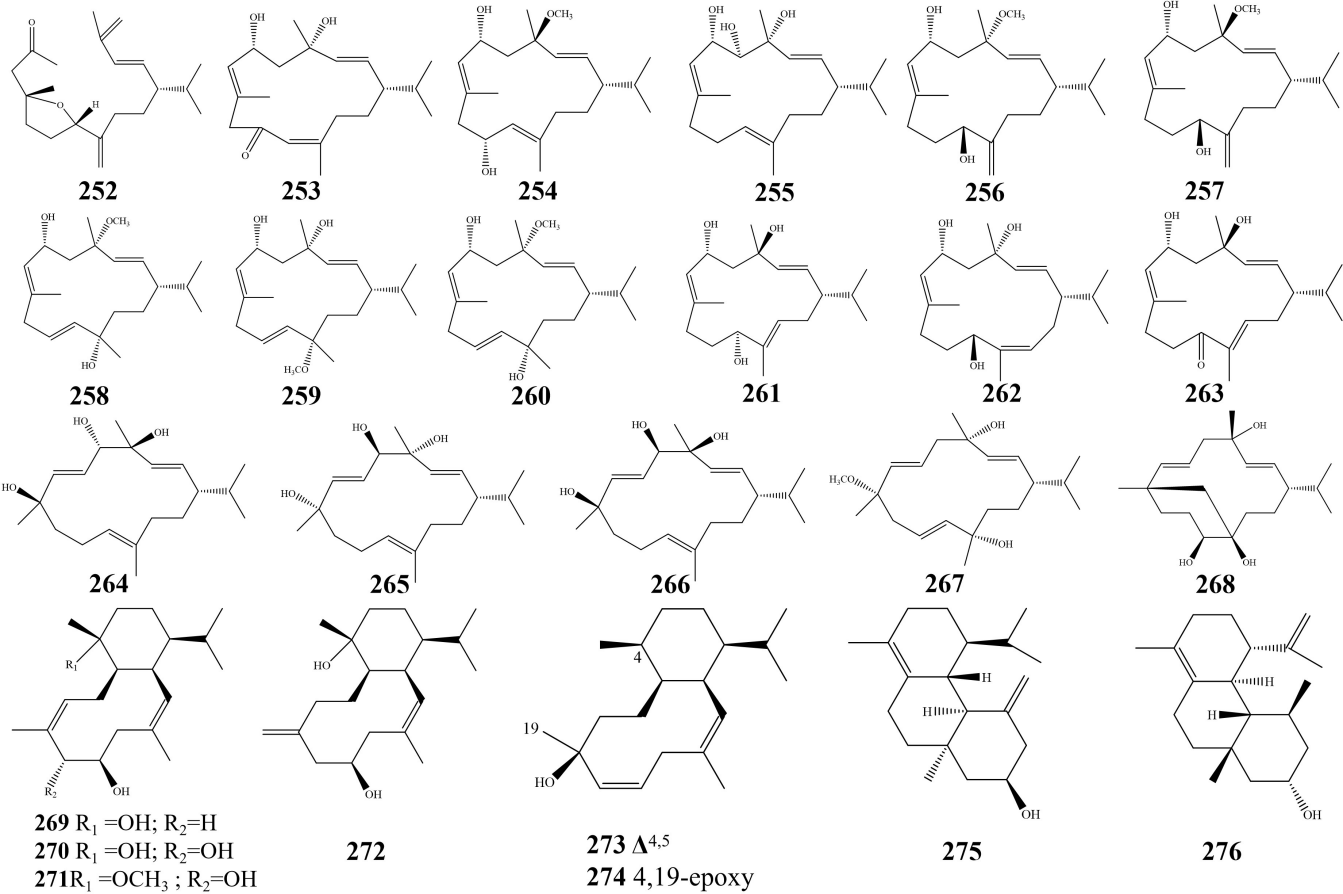
Chemical structures of diterpenes (252−276) isolated from *Nicotiana*.

Diterpenes with a C20 skeleton based on four isoprene units are a structurally diverse class of terpenes. There are more than 126 different diterpene carbon skeletons have been identified (Roncero et al., 2018). Cembrane-type diterpenes are most structurally diverse and prevalent terpenes in cultivated types of tobacco. There are 131 natural cembranoid diterpenes 81-211, including isopropyl cembranoids (1-88), seco-cembranoids (89–103), chain cembranoids (104–123), and polycyclic cembranoids (124–131), reported in tobacco since 1961 (Xu et al., 2024a). Compared to tobacco cembranoids, there have been relatively few studies on natural labdane diterpenes, which are mainly found in oriental tobacco. Xu et al. have summarized and drawn chemical structures of 40 labdane diterpenes 212-251, including epoxy side chain labdanes (132–150) and epoxy-free side chain labdanes (151–171), reported in tobacco since 1961 (Xu et al., 2024a). **Figure 4** lists some previously undescribed cembrane-type diterpenes (252-276) isolated from *Nicotiana.* Yan et al. isolated 16 new cembrane-type diterpenoids, named nicotabacins A-P, from the methanol extract of the leaves of *Nicotiana tabacum* L (Yan et al., 2025). Meanwhile, *zhang* et al. found nine previously undescribed cembrane-type diterpenoids, Cigarcembrane A-I, from Yunnan local cigar tobacco (Zhang et al., 2025).

### 3.4 Triterpenes and tetraterpenes

**Figure 5:**
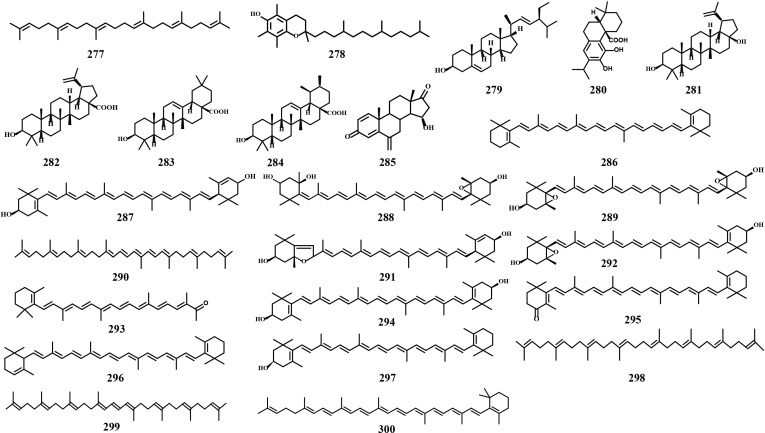
Chemical structures of triterpenes and tetraterpenes (277−300) isolated from *Nicotiana*.

Triterpenoids are a large class of terpenoids characterized by a 30-carbon skeleton. At present, there are few triterpenoid compounds found in tobacco (**Table 1**; **Figure 5**). Popova et al. identified squalene, *α*-tocopherol and *β*-stigmasterol in resinoid from *N. glutinosa* leaves (Popova et al., 2019c). Five triterpenes (carnosic acid, botulin, betulinic acid, oleanolic acid and ursolic acid) were also identified in the leaves of three *Nicotiana* species (Popova et al., 2019b). A new sterol with a cyclopenta[*a*]phenanthrene core was obtained from *N. tabacum* leaves (Shang et al., 2019). Tetraterpenes are C40-compounds consisting of eight isoprene building units and include compounds like carotenoids (responsible for the red, orange, and yellow colors in flowers and vegetables). *Xiao* et al. identified 36 carotenoids in all four cultivars involved in *N. tabacum* and *N. rustica* (Xiao et al., 2022). The major carotenoids in green tobacco tissue are *β*-carotene, lutein, violaxanthin and neoxanthin (Wahlberg and Enzell, 1987).

## 4 Biosynthesis

**Figure 6:**
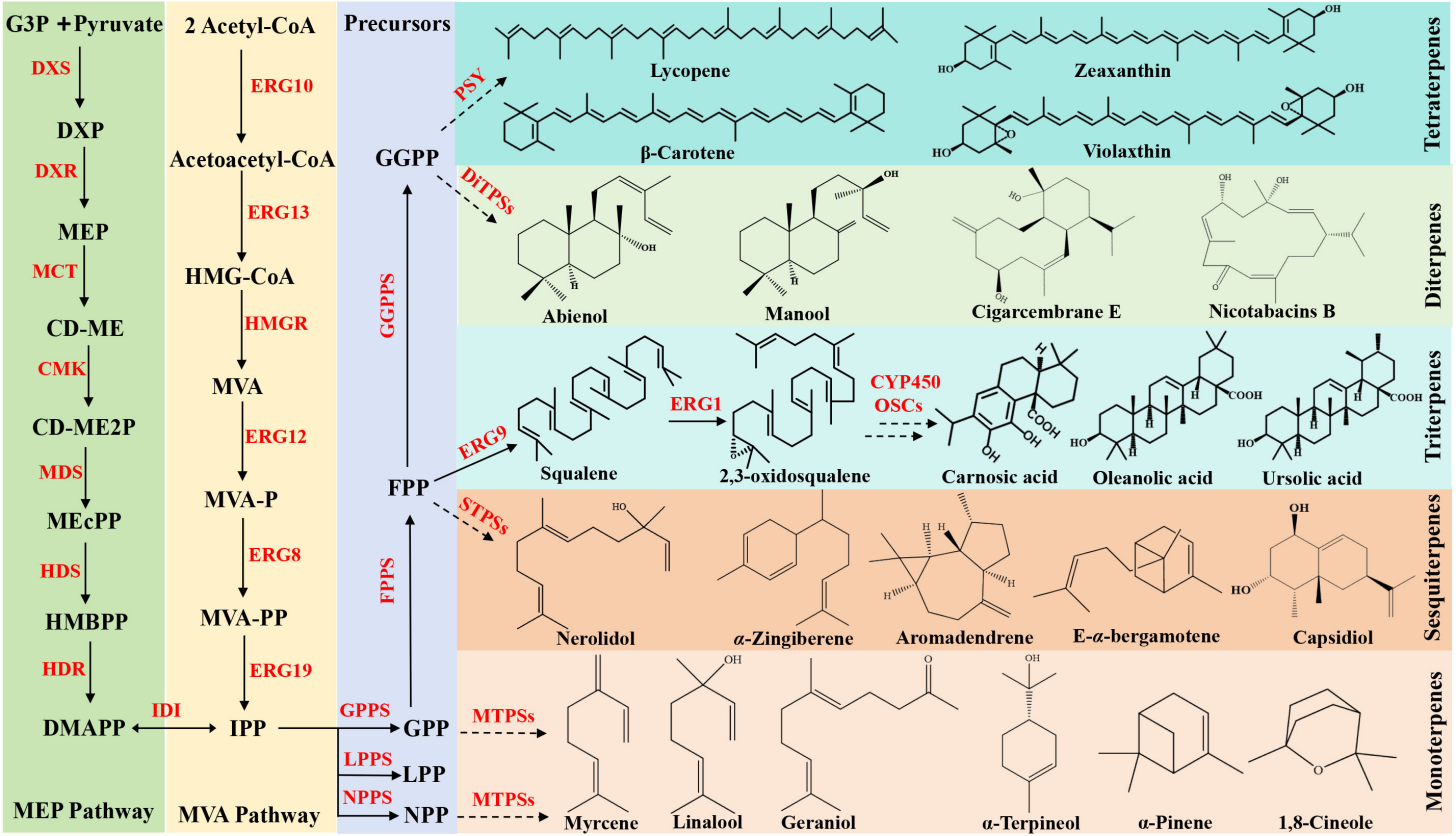
Metabolic pathway of terpenoids biosynthesis. Enzymes are indicated in red text. DXS, 1-deoxy-D-xylulose-phosphate synthase; DXR, 1-deoxy-D-xylulose 5-phosphate reductoisomerase; MCT, 2-C-methyl-D-erythritol-phosphate cytidylyltransferase; CMK, 4- (cytidine-5′-diphospho)-2-C-methyl-D-erythritol kinase; MDS, 2C-methyl-d-erythritol 2, 4-cyclodiphosphate synthase; HDS, 4-hydroxy-3-methylbut-2-enyl diphosphate synthase; HDR, 4-hydroxy-3-methylbut-2-en-1-yl diphosphate reductase; ERG10, acetoacetyl-CoA thiolase; ERG13, 3-hydroxy-3-methylglutaryl-CoA synthase; HMGR, 3-hydroxy-3-methylglutaryl-CoA reductase; ERG12, mevalonate kinase; ERG8, phosphomevalonate kinase; ERG19, mevalonate diphosphate decarboxylase; IDI, isopentenyl diphosphate isomerase; GPPS, geranyl diphosphate synthase; LPPS, lavandulyl diphosphate synthase; NPPS, neryl diphosphate synthase; FPPS, farnesyl diphosphate synthase; GGPPS, geranylgeranyl diphosphate synthase; MTPSs, monoterpene synthases; STPSs, sesquiterpene synthases; DiTPSs, diterpenes synthases; PSY, phytoene synthase; ERG9, squalene synthase; ERG1, squalene monooxygenase; CYP450, cytochrome P450 oxygenases; OSCs, oxidosqualene cyclases.

Terpenoids biosynthesis greatly contributes to the structural diversity of terpene products and intermediates and have been elucidated in various organisms. Broadly, it can be divided into three modules: namely the upstream module for building block formation, midstream module for direct precursors formation, and downstream module for skeletons assembly and post-modification (**Figure 6**) (Andersen et al., 2019).

### 4.1 Building block formation

First, all terpenoids begin with the universal five-carbon (C5) isoprenoids, isopentenyl diphosphate (IPP) and its isomer dimethylallyl diphosphate (DMAPP), which can be produced via the cytosolic mevalonate (MVA) or the plastidial 2-Cmethyl-D-erythritol-4-phosphate (MEP) pathways. Although the MEP and MVA pathways share the same end product, IPP and DMAPP, these pathways differ considerably. The MEP pathway begins with D-glyceraldehyde-3-phosphate (G3P) and pyruvate to yield IPP/DMAPP over a sequence of seven reactions in the plastid. The MVA pathway originates from the Claisen condensation of two acetyl-CoA to produce IPP/DMAPP in six enzymatic steps in the cytoplasm. In addition, the energy and cofactor consumption differs between the two pathways. The MEP pathway consumes one molecule of glucose, three molecules of ATP and two molecules of NAD(P)H for the synthesis of one IPP/DMAPP molecule. However, the MVA pathway requires 1.5 molecules of glucose and produces four molecules of NAD(P)H (Li et al., 2020). Both MVA and MEP pathways conspire through exchange of intermediates and regulatory interactions.

### 4.2 Direct precursors formation

During the second modules of terpenoid biosynthesis, prenyltransferases catalyze consecutive head-to-tail condensation reactions of IPP and DMAPP to form various natural isoprenoids with different chain lengths (Chang et al., 2021). First, the head-to-tail fusion of IPP and DMAPP to geranyl diphosphate (GPP) or neryl diphosphate (NPP) is catalyzed by geranyl diphosphate synthase (GPPS) and neryl diphosphate synthase (NPPS), respectively. While the non–head-to-tail condensation of two DMAPP to lavandulyl diphosphate (LPP) is catalyzed by lavandulyl diphosphate synthase (LPPS) (Demissie et al., 2013). GPP and NPP serve as precursors for regular monoterpenes, while LPP is used as a substrate for irregular monoterpenes. Then, the condensation of one DMAPP and two IPP molecules catalyzed for the synthesis of farnesyl diphosphate (FPP) by the enzyme FPP synthase (FPPS), serving as the backbone of sesquiterpenes. Further elongation via geranyl diphosphate synthase (GGPPS) incorporates an additional IPP with FPP to form geranylgeranyl diphosphate (GGPP), a precursor for diterpenes. On this basis, two molecules of FPP and GGPP are, respectively, head-to-head condensed to form triterpenes and tetraterpenes, respectively.

### 4.3 Skeletons assembly and post-modification

The upstream and midstream modules are universal in the biosynthetic pathway of terpenoids. The downstream module, skeletons assembly and post-modification, are diverse and drastically increases the diversity of terpenoid constituents (Vranová et al., 2013). In this module, terpene synthase (TPS) enzymes further assemble these direct precursors (GPP/NPP/FPP/GGPP) into diverse terpene scaffolds. Monoterpene synthases (MTPSs) remove the bisphosphate groups of the direct precursors GPP or NPP to form monoterpenes skeletons, and sesquiterpene synthases (STPSs) utilize FPP as a substrate to produce sesquiterpenes skeletons, and diterpenes synthases (DiTPSs) convert GGPP into diterpenes skeletons. Two FPP molecules can also be converted into linear C30 compound 2,3-oxidosqualene via squalene synthase (ERG9) and squalene monooxygenase (ERG1). Then, 2,3-oxidosqualene is cyclized by members of oxidosqualene cyclases (OSCs) to form various triterpene skeletons. Phytene synthetase (PSY) can also directly condense two GGPP molecules to form the carotenoid precursor octahydrolycopene. Finally, specific terpene skeletons are further decorated by other groups of enzymes such as cytochrome P450 monooxygenases (P450s) for oxygenation, glycosyltransferases for glycosylation or acyltransferases for acylation.

### 4.4 Key genes

Terpene synthase (TPS) enzymes are the gatekeepers in generating the tremendous variety of terpenoid carbon structures. There are 160 TPS genes identified through sequence analyses of the whole tobacco genome (Rabara et al., 2023). However, only 15 TPSs of tobacco have been cloned and functionally characterized so far (**Table 2**). A chloroplast-localized NtTPS2 utilize GPP as a substrate to produce monoterpenes geraniol and nerol (Liu et al., 2021). A cytosol-localized NtTPS25 can also convert GPP into the monoterpene E-*β*-ocimene (Li, 2021). The cineole synthase (CIN) of *N. noctiflora* catalyzes the cyclization of GPP into the seven monoterpenes of the ‘cineole cassette’: *α*-pinene, *β*-pinene, sabinene, *β*-myrcene, limonene, 1,8-cineole and *α*-terpineol (Fähnrich et al., 2014, 2012). There are five sesquiterpene synthases functionally characterized by experimental means. 5-epi-aristolochene synthase (EAS) from *N. tabacum* converts FPP into 5-epi-aristolochene. In 1997, the crystal structures of EAS and its complexed separately with two FPP analogs have been analyzed (Starks et al., 1997). NtTPS7 and NtTPS21 can both catalyze the synthesis of sesquiterpene *β*-caryophyllene (Cheng et al., 2022; Yang et al., 2022). NtTPS126 utilize FPP as a substrate to produce *β*-longipinene as the main product and *α*-eudesmol as side products (Yang et al., 2024a). NaTPS38 converted the substrate (E,E)-FPP into (E)-*α*-bergamotene as the sole product (Zhou et al., 2017). *Sallaud*. et al. has cloned and characterized two genes from tobacco, NtCPS2 and NtABS, necessary and sufficient for *Z*-abienol biosynthesis from GGPP in glandular trichomes. NtCPS2 encodes a class-II terpene synthase that synthesizes 8-hydroxy-copalyl diphosphate, and NtABS encodes a kaurene synthase-like protein that uses 8-hydroxy-copalyl diphosphate to produce *Z*-abienol (Sallaud et al., 2012). For cembratrien-diols synthesis, NtCBTS encoded cembratrien-ol synthases have also identified in *N. tabacum* (Ennajdaoui et al., 2010). *Yang*. et al. have identified and biochemically characterized 2 key oxidosqualene cyclases (OSCs) of *N. attenuata.* NaOSC1is a multifunctional enzyme capable of synthesizing lupeol, dammarenediol II, 3-alpha,20-lupanediol, and 7 other triterpene scaffolds, and NaOSC2 is a selective enzyme, producing only the *β*-amyrin scaffold (Yang et al., 2023). Cytochrome P450 enzymes (P450s) are versatile biocatalysts that play critical roles in terpenoid skeleton modification and structural diversity. A total of 142 NtCYP450 genes were identified in the tobacco genome (Sha et al., 2026), while only five P450s have been verified to be involved in terpenoids biosynthesis (**Table 2**). CYP71D20, also termed 5-epi-aristolochene dihydroxylase (EAH), is responsible for the conversion of 5-epi-aristolochene to capsidiol (Ralston et al., 2001). The trichome-specific CYP71D16 converts diterpene alcohols *α/β*-cembratrien-ol to *α/β*-cembratrien-diol, respectively (Wang et al., 2001). Another three P450s, NaCYP716A419, NaCYP716E107 and NaCYP716C87, can catalyze the oxidation of *β*-amyrin, lupeol, lupanediol, or their downstream compound skeletons at C28, C6*β*, and C2*α* positions, respectively (Yang et al., 2024b). In *N. tabacum*, carotenoids can be degraded into many aroma substances. There are two primary enzymatic pathways mediated by carotenoid cleavage dioxygenases (CCDs) and lipoxygenases (LOXs). CCDs can catalyze the oxidative cleavage of carotenoids to yield apocarotenoids, including *β*-ionone, geranylacetone, pseudoionone and *α*-ionone. *Liu*. et al. have demonstrated that NtCCD1/4/7 effectively could catalyze the cleavage of *β*-carotene to produce *β*-ionone (Liu et al., 2026). In addition, NtCCD10 could symmetrically cleave phytoene and *β*-carotene at the C9–C10 and C9’–C10’ positions to produce geranylacetone and β-ionone, respectively (Li et al., 2022). LOXs catalyze the co-oxidation reaction of carotenoids to generate key flavor volatiles, including *β*-ionone, *β*-cyclocitral, *β*-ionone-5,6-epoxide, and dihydroactinidiolide. *Dong*. et al. demonstrate that NtLOX2 mediates the co-oxidation of carotenoid including *β*-carotene, lutein, violaxanthin, and neoxanthin, leading to enhanced accumulation of volatile aroma compounds in tobacco leaves (Dong et al., 2026). Besides, the synthesis and accumulation of terpenoids in tobacco plants are spatiotemporally regulated by a complex network in which transcription factors play a pivotal role. NtERF10, a AP2/ERF transcription factor, can positively regulate some key genes related to the terpene synthesis pathway (Xu et al., 2024b). NtPIF1 (a PIF transcription factor), NtDREB-1BL1 (a dehydration-responsive element-binding protein (DREB) transcription factor), and NtCYC (the teosinte branched 1/CYCLOIDEA/PCF (TCP) transcription factor) can regulate carotenoids biosynthesis in tobacco (Dong et al., 2022; Liu et al., 2023b; Li et al., 2025). NtWHY1, a whirly transcription factor, positively regulates the cembranoid diterpenoids biosynthesis by directly targeting NtCBTS in tobacco (Zhai et al., 2025).

## 5 Engineering terpenes metabolism in tobacco

Because tobacco is amenable to *Agrobacterium-*mediated transient expression, many biosynthetic pathways of structurally complex terpenes, including baccatin III (Jiang et al., 2024a) and astragalosides (Xu et al., 2024c), have been reconstructed in tobacco plants, specifically *Nicotiana benthamiana*. And moreover, the terpenes metabolism in tobacco have been also widely modified by several metabolic engineering strategies (**Table 3**). In generally, these engineering strategies to enhance the yield of desired terpenes natural products in tobacco can be considered from the two aspects: host engineering and enzyme engineering.

**Table 3:** Representative examples of engineering terpenes metabolism in tobacco.

Compound	Class	Strategies	Quantity	Fold increases	Phenotypes	References
Limonene	Monoterpene	Expression of *Mentha × piperita* geranyl diphosphate synthase small subunit	9.56 µg/g FW	22~35-fold	Early flowering and shoot branching increasing	(Yin et al., 2017)
Linalool	Monoterpene	Expression of lily terpene synthase	_	2~3-fold	_	(Zhang et al., 2020)
Bergamotene	Sesquiterpene	Chloroplast targeting and cytosolic MVA pathway enhancement	268.6 ng/g FW	500 ~ 1000-fold	More attractive to green peach aphids	(Yin and Wong, 2019)
*β*-Caryophyllene	Sesquiterpene	Introducing oleosin-coated lipid bodies	_	2~4-fold	_	(Delatte et al., 2018)
*β*-Caryophyllene	Sesquiterpene	Silencing of VAMP72 genes by RNAi strategy	_	5-fold	_	(Ting et al., 2015)
Valencene	Sesquiterpene	Simultaneous silencing of SQS and EAS by RNAi strategy	_	2.8-fold	_	(Cankar et al., 2015
Sclareol	Diterpene	Expression of *Salvia sclarea* labda-13-en-8-ol diphosphate synthase and sclareol synthase	4.1 μg/cm^2^	3.4-fold	_	(Ma et al., 2024)
Cembratrien-ols	Diterpene	Expression of *Nicotiana tabacum* cembratrien-ol synthase 2 gene	0.96 μg/cm^2^	2~3-fold	Promoting aphid resistance	(Zhang et al., 2018)
Taxadiene 5α-hydroxylase	Diterpene	Tuning the promoter strength	–	3-fold	–	(Liu et al., 2024)
Ursolic acid	Triterpene	Applying “Tsukuba system”	33.92 mg /g DW	1.65-fold	_	(Romsuk et al., 2024)
*α*-Tocopherol	Triterpene	Introducing stress inducible promoters of arabidopsis	0.6 μmol/g FW	10-fold	Alleviating stress-induced leaf damage	(Espinoza et al., 2013)
Carotenoid	Tetraterpene	Rational design of geranylgeranyl diphosphate synthase	_	_	Improving drought tolerance	(Song et al., 2025)

### 5.1 Host engineering

Increasing precursor supply is a common strategy to increase the titer of final products. Engineering strategies to increase terpenes production have focused on the gene overexpression of related enzymes in the MEP or MVA pathway. For example, DXS is the first rate-limiting enzyme involved in the MEP pathway for terpenoids biosynthesis. Brückner et al. found that co-expression of DXS and GGPPS with CBTS resulted in a significant 3.5-fold increase in the production of CBT-ol in *N. benthamiana* (Brückner and Tissier, 2013). For MVA pathway, HMGR is known to be a key rate-limiting enzyme. Overexpressing the genes of truncated HMGR led to the production of higher amounts of santalenes and bergamotene in transgenic tobacco plants (Yin and Wong, 2019). Suppression of unwanted competitive metabolic pathways can also effectively direct metabolic flux toward the synthesis of terpenes compounds. FPP is an important intermediate in terpenoid biosynthesis, which can be converted to aristolochene by endogenous 5‐epi‐aristolochene synthase (EAS), or squalene by squalene synthase (SQS) in *N. benthamiana*. Simultaneous silencing of endogenous SQS and EAS by RNAi strategy resulted in a 2.8‐fold increase of valencene content in transgenic *N. benthamiana* expressing heterogenous valencene synthase (Cankar et al., 2015). Terpenes are stored in vesicles which are transported to the plasma membrane and VAMP72 protein was shown to mediate the fusion of vesicles with target membranes. Headspace analysis of the leaves showed that caryophyllene emission increased about 5-fold when *N. benthamiana* VAMP72 function was blocked (Ting et al., 2015).

### 5.2 Enzyme engineering

Introducing heterogenous TPSs into tobacco is a common strategy to optimize terpenes natural product production. Heterologous transformation of the *Liriodendron tulipifera* LtuTPS32 into tobacco significantly elevates the levels of chlorophyll, carotenoids, and gibberellins (Wu et al., 2024). Another alternative strategy to engineering terpene production in *N. benthamiana* is to alter the subcellular location of expressed protein by the addition, removal, or modification of target peptides. Dong et al. systematically targeted geraniol synthase and geranyl-diphosphate synthase to each compartment and found targeting to the plastids resulted in the highest levels of geraniol and derivatives, followed by mitochondrial and cytosolic targeting (Dong et al., 2015). In addition, promoter engineering is also a powerful technique to maximize the terpenes production by tuning specific gene expression at the transcriptional level. Homogentisate phytyltransferase (HPT) catalyzes the prenylation step in tocopherol biosynthesis. Transgenic tobacco plants expressing HPT under the control of stress-inducible promoters showed increased levels of *α*-tocopherol when exposed to drought conditions (Espinoza et al., 2013). The pMALD1 promoter, a newly identified trichome-specific promoter, was used to drive the expression of five key genes (farnesyl-diphosphate synthase, squalene synthase, squalene epoxidase, *β*-amyrin synthase and *β*-amyrin 28-monooxygenase), achieving the biosynthesis of triterpenic acids in *N. tabacum* glandular trichomes (Gossart et al., 2023). By tuning the promoter strength for taxadiene 5*α*-hydroxylase expression in *Nicotiana* plants, the accumulation of taxadien-5*α*-ol was increased by three-fold (Liu et al., 2024). Rational design and directed evolution for optimizing the performance of each enzyme in the pathway is crucial in metabolic engineering. Song et al. found that rational design of geranylgeranyl diphosphate synthase1 from *N. tabacum* (NtGGPPS1) enhanced carotenoid biosynthesis and increased its drought tolerance (Song et al., 2025).

## 6 Ecological functions

**Figure 7:**
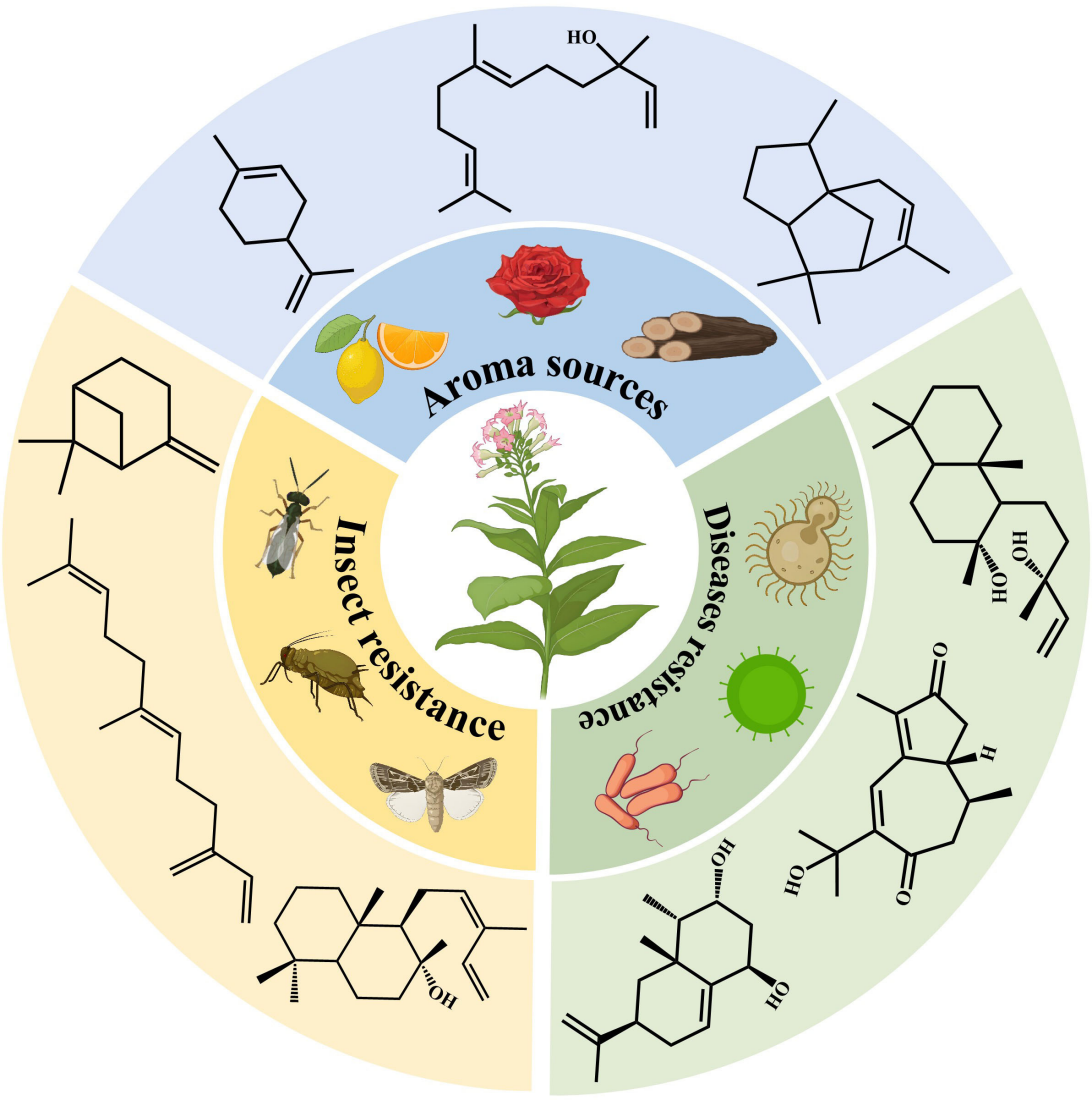
Ecological functions of the terpenes in tobacco. Created with BioRender.com.

Terpenoids are among the most significant components of volatile organic compounds in tobacco and volatile terpenoids directly influence the flavor and fragrance of tobacco products. In addition, terpenoids play several physiological and ecological functions in tobacco life, responding to environmental changes, and combating pathogenic microorganisms, pests, and diseases (**Figure 7**).

### 6.1 Aroma sources

Tobacco aroma is an important quality attribute of tobacco and is dependent on its aroma compounds, among which are terpenoids (Jiang et al., 2024b). Monoterpenes and sesquiterpenes are the main chemical characteristic constituents of volatile organic compounds, which are responsible for imparting specific aromas to tobacco. Geraniol, linalool, nerol, (+)-limonene and *α*-terpineol are the most important odor-active monoterpenoids and contribute to the varietal aroma profiles of tobacco due to their lemon-like and citrus-like fruity odors. Farnesol and (S)-nerolidol are examples of sesquiterpenoids that are associated with a flowery scent (Sommer et al., 2022). *β-*Caryophyllene, *cis*-*β*-farnesene, *α*-humulene and *β*-selinene exhibited herbal, green, spicy and woody odors (Jiang et al., 2024b). Although the volatile monoterpenes and sesquiterpenes are considered to be the main contributors of cigarette flavor due to their direct influence on the senses, diterpenes and carotenoids are also are important aroma precursors for flavor formation during the tobacco curing process. Abienol, a polycyclic labdane-related diterpenoid, is a precursor of tobacco amber-like compounds that form during leaf curing and impact upon smoke quality. To the best of our knowledge, tobacco plants contain the highest content of cembranoid diterpenes, which are significant flavor precursors found in flue-cured tobacco leaves. Their degradation during the aging and curing processes is closely related to the aroma quality of tobacco (Cai et al., 2017). In addition, tobacco contains over a hundred carotenoid degradation products, which possess appealing aromas (Meng et al., 2024). For example, *β*-ionone exudes a violet scent, geranyl acetone evokes a magnolia aroma, and megastigmatrienone emits tobacco-like aromas. Besides, carotenoid degradation products have been demonstrated to mellow the smoke and reduce irritation. The addition of *β*-ionone to tobacco significantly enhances the aroma quality of cured tobacco leaves, while reducing harshness and irritancy (Liu et al., 2022).

### 6.2 Insect resistance

In tobacco plants, terpenoids exhibit remarkable efficacy in deterring herbivorous insects, operating through both volatile and non-volatile mechanisms. Volatile terpenoids, particularly monoterpenes and sesquiterpenes, act as an indirect line of defense (Jassbi et al., 2017). When *N. attenuate* is attacked by specific herbivores like *Manduca sexta*, there is a notable increase in the emission of volatile monoterpenes and sesquiterpenoids (Zhou et al., 2017). In tobacco planting fields, the synthetic mixture of monoterpenoids (*β*-pinene, methyl salicylate, linalool, and limonene) significantly increased the population of natural enemies of aphids (Wu et al., 2022). Previous study showed most pure monoterpenoid compounds could exhibit acute or sublethal growth effects against the tobacco cutworm (*Spodoptera litura Fab*.) (Hummelbrunner and Isman, 2001), and numerous studies suggest that monoterpenes primarily exert their insecticidal effects by targeting the nervous system of insects (Oliveira et al., 2024). Notably, limonene has been registered as a pesticide, a fungicide, and a pesticide adjuvant in China (Luo et al., 2023). Additionally, volatile sesquiterpenoids like trans-*α*-bergamotene and trans-*β*-farnesene can attract predators like big-eyed bugs (*Geocoris pallens*), which effectively control herbivore populations (Kessler and Baldwin, 2001). *β*-Farnesene can mediate interactions between plants and aphids as both an alarm pheromone produced by aphids and a semiochemical produced by plants to attract aphid predators (Wang et al., 2022). Wang. et al. expressed an (E)-*β*-farnesene synthase in the chloroplast of tobacco, which enhanced repellence to green peach aphid (*Myzus persicae*) and attracted response of its parasitoid *Diaeretiella rapae* (Wang et al., 2015).

In contrast, non-volatile terpenoids offer a different mode of protection. Studies demonstrated that the insecticidal effects of *N. tabacum* cultivars were strongly related to their concentration of leaf exudate metabolites, and in particular to diterpenes (sclareol, labdenediol, cis-abienol, 13-epi-sclareol, etc.) (Simpson et al., 1985). For example, *cis*-Abienol is the most abundant labdane diterpenoid in tobacco trichome secretions and is involved in insect resistance. Furthermore, cembratrien-ol (CBT-ol) isolated from tobacco reportedly showed excellent insecticidal activity (Yan et al., 2019). Overexpression of cembratrien-ol synthase gene greatly increased aphid resistance by promoting the accumulation of CBT-ols in tobacco plants (Zhang et al., 2018). Mischko et al. further confirmed the insecticide characteristics of CBT-ol *in vivo* and *in vitro* bioactivity studies (Mischko et al., 2018).

### 6.3 Diseases resistance

Previous phytochemical findings on *N. tabacum* showed terpenoids play also a crucial role in defending the plants themselves against diseases (Zhang et al., 2024). Terpenoids have demonstrated the ability to counter tobacco mosaic virus (TMV) infection (Bailey et al., 1975; Shang et al., 2016). NtSPS1 functions as a key enzyme in the biosynthetic pathway of solanesol (Campbell et al., 2016). Dai. et al. found the solanesol content and anti-TMV properties were remarkable downregulation in *NtSPS1* knockout tobacco (Dai et al., 2025). Luo. et al. demonstrated that limonene also exhibited excellent anti-TMV bioactivity (Luo et al., 2023). When infected by TMV, capsidiol (a sesquiterpenoid phytoalexin) is produced in *N. tabacum*, suggesting that capsidiol may play a role in TMV resistance (Nugroho et al., 2002). Thymol was also noted to be powerful agents against the TMV (Astani et al., 2010).In addition, previous published work have reported that the presence of cembranoids in tobacco plants could exhibit inhibitory activities on fungal pathogens, including *Alternaria alternata*, *Aspergillus niger*, *Candida albicans*, *Fusarium chlamydosporum* (Guan et al., 2023; Yan et al., 2019). Two guaiane-type sesquiterpenoids, nicotiasesquiterpenes A and B, showed also obvious anti-phytopathogenic fungal activity against *Valsa mali* var. *mali*, *Alternaria porri*, and *Botrytis cinerea* (Xu et al., 2022). Furthermore, many terpenoids in tobacco plants such as cembratriene-4,6-diols (CBT-diols) and sclareol, had been known to exert antimicrobial activity against a wide variety of bacteria. For example, CBT-diols have antibacterial activities against *Bacillus subtilis*, *Staphylococcus aureus*, and *Proteus vulgaris* (Aqil et al., 2011). When exogenously applied to their roots, sclareol and cis-abienol inhibited wilt disease in tobacco (Seo et al., 2012).

## 7 Conclusions

To date, studies on tobacco terpenes have revealed that these compounds are of great significance to tobacco aromas and play several physiological and ecological functions in tobacco life. This review is the first systematic summary of the chemical structures, biosynthesis, metabolic engineering and ecological functions for terpenes produced in tobacco plants. About 300 reported natural tobacco terpenes collected from published studies were summarized and classified based on the number of these isoprene units. A brief overview of the terpene biosynthesis, endogenous terpene synthases and metabolic strategies for terpenes in tobacco was carried out. A comprehensive review of ecological functions, including contributing to tobacco’s distinctive aroma, enhancing tobacco resistance to insect and diseases, has also been discussed.

This review shows that tobacco terpenes have attracted more and more attention due to their diverse chemical structure and prominent ecological functions. Although a large number of terpenes have been characterized from *N. tabacum*, their phytochemical investigations and biological activities have not been fully studied due to low yields and complex purification procedures. Future research should place emphasis on produce high-purity terpenoids by means of synthetic biology and explore the structure-property relationships tobacco terpenes to lay a foundation for their application in pharmaceutical and agrochemical purposes. The terpenoid biosynthesis is tissue-specific regulatory network and tightly linked to plant responses under stress. Some key genes are only transiently expressed in specific tissue or activated under specific stress conditions. So, the detailed genes and pathways involved in biosynthesis of different tobacco terpenes are still not fully elucidated. Further studies should also focus on characterizing more unknown terpene synthase and elucidating biosynthesis pathways of tobacco terpenes.

## References

[B1] Andersen T. B. Rasmussen S. A. Christensen S. B. Simonsen H. T. (2019). Biosynthesis of tovarol and other sesquiterpenoids in Thapsia laciniata Rouy. Phytochemistry 157, 168–174. doi: 10.1016/j.phytochem.2018.10.027 30412824

[B2] Aqil F. Zahin M. El Sayed K. A. Ahmad I. Orabi K. Y. Arif J. M. (2011). Antimicrobial, antioxidant, and antimutagenic activities of selected marine natural products and tobacco cembranoids. Drug Chem. Toxicol. 34, 167–179. doi: 10.3109/01480545.2010.494669 21314466

[B3] Astani A. Reichling J. Schnitzler P. (2010). Comparative study on the antiviral activity of selected monoterpenes derived from essential oils. Phytother. Res. 24, 673–679. doi: 10.1002/ptr.2955 19653195 PMC7167768

[B4] Bailey J. A. Carter G. A. Burden R. S. Wain R. L. (1975). Control of rust diseases by diterpenes from Nicotiana glutinosa. Nature 255, 328–329. doi: 10.1038/255328a0 37880705

[B5] Berenguer C. Pereira J. A. M. Câmara J. S. (2021). Fingerprinting the volatile profile of traditional tobacco and e-cigarettes: A comparative study. Microchem. J. 166, 106196. doi: 10.1016/j.microc.2021.106196 38826717

[B6] Brückner K. Tissier A. (2013). High-level diterpene production by transient expression in Nicotiana benthamiana. Plant Methods 9, 46. doi: 10.1186/1746-4811-9-46 24330621 PMC3878842

[B7] Cai K. Xiang Z. Li H. Zhao H. Lin Y. Pan W. . (2017). Free amino acids, biogenic amines, and ammonium profiling in tobacco from different geographical origins using microwave‐assisted extraction followed by ultra-high performance liquid chromatography. J. Sep. Sci. 40, 4571–4582. doi: 10.1002/jssc.201700608 29131486

[B8] Campbell R. Freitag S. Bryan G. J. Stewart D. Taylor M. A. (2016). Environmental and genetic factors associated with solanesol accumulation in potato leaves. Front. Plant Sci. 7, 1263. doi: 10.3389/fpls.2016.01263 27610114 PMC4996988

[B9] Cankar K. Jongedijk E. Klompmaker M. Majdic T. Mumm R. Bouwmeester H. . (2015). (+)-Valencene production in Nicotiana benthamiana is increased by down-regulation of competing pathways. Biotechnol. J. 10, 180–189. doi: 10.1002/biot.201400288 25159317

[B10] Chang H. Cheng T. Wang A. H. (2021). Structure, catalysis, and inhibition mechanism of prenyltransferase. IUBMB Life 73, 40–63. doi: 10.1002/iub.2418 33246356 PMC7839719

[B11] Chen Y. K. Meng L. C. Zhong B. S. Wang L. Yang G. Y. Miao M. M. (2014). A new sesquiterpene from Nicotiana tabacum and its cytotoxicity. Asian J. Chem. 26, 2246–2248. doi: 10.14233/ajchem.2014.15680

[B12] Cheng T. Zhang K. Guo J. Yang Q. Li Y. Xian M. . (2022). Highly efficient biosynthesis of β-caryophyllene with a new sesquiterpene synthase from tobacco. Biotechnol. Biofuels Bioprod. 15, 39. doi: 10.1186/s13068-022-02136-8 35468840 PMC9040381

[B13] Christianson D. W. (2017). Structural and chemical biology of terpenoid cyclases. Chem. Rev. 117, 11570–11648. doi: 10.1021/acs.chemrev.7b00287 28841019 PMC5599884

[B14] Couto M. A. M. S. Soares G. L. G. Turchetto C. (2024). Exploring floral scent in wild tobacco: comparison of volatile compounds across pollinator functional groups and Nicotiana sections. Evol. Ecol. 38, 409–432. doi: 10.1007/s10682-024-10301-8 30311153

[B15] Dai J. M. Zhang J. D. Liu X. Zhang L. F. Wang J. Xu Y. . (2025). Gene editing, metabolomics, network pharmacology strategies to explore terpenoid content and anti-TMV activity in NtSPS1 knockout Nicotiana tabacum. Sci. Rep. 15, 14581. doi: 10.1038/s41598-025-98745-y 40280998 PMC12032281

[B16] Delatte T. L. Scaiola G. Molenaar J. de Sousa Farias K. Alves Gomes Albertti L. Busscher J. . (2018). Engineering storage capacity for volatile sesquiterpenes in Nicotiana benthamiana leaves. Plant Biotechnol. J. 16, 1997–2006. doi: 10.1111/pbi.12933 29682901 PMC6230952

[B17] Demissie Z. A. Erland L. A. E. Rheault M. R. Mahmoud S. S. (2013). The biosynthetic origin of irregular monoterpenes in Lavandula. J. Biol. Chem. 288, 6333–6341. doi: 10.1074/jbc.M112.431171 23306202 PMC3585068

[B18] Dong C. Song L. Li F. Wang B. Wang X. Chen D. . (2026). Lipoxygenase 2 (LOX2) coordinates carotenoid and methyl jasmonate metabolism in Nicotiana tabacum. Biochem. Biophys. Res. Commun. 808, 153466. doi: 10.1016/j.bbrc.2026.153466 41713197

[B19] Dong C. Wang Q. Wang Y. Qin L. Shi Y. Wang X. . (2022). NtDREB-1BL1 enhances carotenoid biosynthesis by regulating phytoene synthase in nicotiana tabacum. Genes 13, 1134. doi: 10.3390/genes13071134 35885917 PMC9322988

[B20] Dong L. Jongedijk E. Bouwmeester H. Krol A. V. D. (2015). Monoterpene biosynthesis potential of plant subcellular compartments. New Phytol. 209, 679–690. doi: 10.1111/nph.13629 26356766

[B21] Ellis M. D. Hoak J. M. Ellis B. W. Brown J. A. Sit T. L. Wilkinson C. A. . (2020). Quantitative real-time PCR analysis of individual flue-cured tobacco seeds and seedlings reveals seed transmission of tobacco mosaic virus. Phytopathology 110, 194–205. doi: 10.1094/PHYTO-06-19-0201-FI 31502520

[B22] Ennajdaoui H. Vachon G. Giacalone C. Besse I. Sallaud C. Herzog M. . (2010). Trichome specific expression of the tobacco (Nicotiana sylvestris) cembratrien-ol synthase genes is controlled by both activating and repressing cis-regions. Plant Mol. Biol. 73, 673–685. doi: 10.1007/s11103-010-9648-x 20495852

[B23] Espinoza A. San Martín A. López-Climent M. Ruiz-Lara S. Gómez-Cadenas A. Casaretto J. A. (2013). Engineered drought-induced biosynthesis of α-tocopherol alleviates stress-induced leaf damage in tobacco. J. Plant Physiol. 170, 1285–1294. doi: 10.1016/j.jplph.2013.04.004 23651908

[B24] Fähnrich A. Brosemann A. Teske L. Neumann M. Piechulla B. (2012). Synthesis of ‘cineole cassette’ monoterpenes in Nicotiana section Alatae: gene isolation, expression, functional characterization and phylogenetic analysis. Plant Mol. Biol. 79, 537–553. doi: 10.1007/s11103-012-9933-y 22669744

[B25] Fähnrich A. Krause K. Piechulla B. (2011). Product variability of the “cineole cassette” monoterpene synthases of related Nicotiana species. Mol. Plant 4, 965–984. doi: 10.1093/mp/ssr021 21527560

[B26] Fähnrich A. Neumann M. Piechulla B. (2014). Characteristic alatoid ‘cineole cassette’ monoterpene synthase present in Nicotiana noctiflora. Plant Mol. Biol. 85, 135–145. doi: 10.1007/s11103-014-0176-y 24493662

[B27] Fei H. A. N. Jinchang L. Hancheng W. Jiangzhou L. I. Jian G. U. O. Ning J. . (2025). Innovation and practices of green control for tobacco diseases in China. Chin. Tob. Sci. 46, 109–117. doi: 10.13496/j.issn.1007-5119.2025.03.013

[B28] Feng T. Li X. M. He J. Ai H. L. Chen H. P. Li X. N. . (2017). Nicotabin A, a sesquiterpenoid derivative from Nicotiana tabacum. Org. Lett. 19, 5201–5203. doi: 10.1021/acs.orglett.7b02559 28880562

[B29] Forestier E. C. F. Brown G. D. Harvey D. Larson T. R. Graham I. A. (2021). Engineering production of a novel diterpene synthase precursor in nicotiana benthamiana. Front. Plant Sci. 12, 757186. doi: 10.3389/fpls.2021.757186 34745188 PMC8564105

[B30] Gossart N. Berhin A. Sergeant K. Alam I. André C. Hausman J. F. . (2023). Engineering Nicotiana tabacum trichomes for triterpenic acid production. Plant Sci. An. Int. J. Exp. Plant Biol. 328, 111573. doi: 10.1016/j.plantsci.2022.111573 36563941

[B31] Guan J. Du Z. Tian T. Wang W. Ju F. Lin X. . (2023). Manipulation of CBTS1 expression alters tobacco resistance to Spodoptera frugiperda and Phytophthora nicotianae. Agronomy 13, 845. doi: 10.3390/agronomy13030845 30654563

[B32] Gui Z. Q. Yuan X. L. Yang J. Du Y. M. Zhang P. (2024). An updated review on chemical constituents from Nicotiana tabacum L.: Chemical diversity and pharmacological properties. Ind. Crops Prod. 214, 118497. doi: 10.1016/j.indcrop.2024.118497 38826717

[B33] He L. X. Liu H. Cheng C. Xu M. He L. Li L. . (2021). RNA sequencing reveals transcriptomic changes in tobacco (Nicotiana tabacum) following NtCPS2 knockdown. BMC Genomics 22, 467. doi: 10.1186/s12864-021-07796-8 34162328 PMC8220664

[B34] Hu R. Shang S. Z. Zhao W. Chen Y. K. Yang G. Y. Liu Z. H. (2015). A new eremophilane-type sesquiterpene from flue-cured tobacco and its anti-tobacco mosaic virus activity. Asian J. Chem. 27, 1947–1948. doi: 10.14233/ajchem.2015.17381

[B35] Hummelbrunner L. A. Isman M. B. (2001). Acute, sublethal, antifeedant, and synergistic effects of monoterpenoid essential oil compounds on the tobacco cutworm, Spodoptera litura (Lep. Noctuidae). J. Agric. Food. Chem. 49, 715–720. doi: 10.1021/jf000749t 11262018

[B36] Jassbi A. R. Zare S. Asadollahi M. Schuman M. C. (2017). Ecological roles and biological activities of specialized metabolites from the genus nicotiana. Chem. Rev. 117, 12227–12280. doi: 10.1021/acs.chemrev.7b00001 28960061

[B37] Jiang B. Gao L. Wang H. Sun Y. Zhang X. Ke H. . (2024a). Characterization and heterologous reconstitution of Taxus biosynthetic enzymes leading to baccatin III. Science 383, 622–629. doi: 10.1126/science.adj3484 38271490

[B38] Jiang C. Lv J. Ji L. An H. Yang M. Huang Y. . (2024b). Characterization of the key aroma compounds in cigar filler tobacco leaves from different production regions. Front. Plant Sci. 15, 1476807. doi: 10.3389/fpls.2024.1476807 39737372 PMC11683104

[B39] Kessler A. Baldwin I. T. (2001). Defensive function of herbivore-induced plant volatile emissions in nature. Science 291, 2141–2144. doi: 10.1126/science.291.5511.2141 11251117

[B40] Kopa P. N. Pawliczak R. (2020). Menthol additives to tobacco products. Reasons for withdrawing mentholated cigarettes in European Union on 20th may 2020 according to tobacco products directive. Toxicol. Mech. Methods 30, 555–561. doi: 10.1080/15376516.2020.1805662 32746758

[B41] Li Z. Y. (2021). Analysis of aroma metabolic pathways and fine mapping of rose aroma genes in tobacco. doi: 10.27630/d.cnki.gznky.2021.001005

[B42] Li F. Gong X. Liang Y. Peng L. Han X. Wen M. (2022). Characteristics of a new carotenoid cleavage dioxygenase NtCCD10 derived from Nicotiana tabacum. Planta 256, 100. doi: 10.1007/s00425-022-04013-y 36251100

[B43] Li M. Hou F. Wu T. Jiang X. Li F. Liu H. . (2020). Recent advances of metabolic engineering strategies in natural isoprenoid production using cell factories. Nat. Prod. Rep. 37, 80–99. doi: 10.1039/C9NP00016J 31073570

[B44] Li J. Liao Q. Zhou H. Hu R. Li Y. Hu Z. . (2025). Multi-omics analyses reveal regulatory networks underpinning metabolite biosynthesis in Nicotiana tabacum. Nat. Commun. 16, 10339. doi: 10.1038/s41467-025-65299-6 41285720 PMC12644487

[B45] Liu T. Cao J. M. Guo C. Gang C. Chen G. Wen L. . (2023a). Difference analysis on chemical composition and aroma components of different flue-cured tobacco cultivars. Chin. Tob. Sci. 44, 74–82. doi: 10.13496/j.issn.1007-5119.2023.02.011

[B46] Liu J. C. De La Peña R. Tocol C. Sattely E. S. (2024). Reconstitution of early paclitaxel biosynthetic network. Nat. Commun. 15, 1419. doi: 10.1038/s41467-024-45574-8 38360800 PMC10869802

[B47] Liu Y. Guo Q. Ji X. Dong A. Fu B. Zhou B. (2022). Preparation and cigarette flavoring application of Megastigmatrienone-β- cyclodextrin inclusion complex. J. Shaanxi Univ. Sci. Technol. 40, 94–99. doi: 10.19481/j.cnki.issn2096-398x.2022.04.017

[B48] Liu S. Zhang Y. Pan X. Li B. Yang Q. Yang C. . (2023b). PIF1, a phytochrome-interacting factor negatively regulates drought tolerance and carotenoids biosynthesis in tobacco. Int. J. Biol. Macromol. 247, 125693. doi: 10.1016/j.ijbiomac.2023.125693 37419268

[B49] Liu W. X. Zhang Z. X. Yang J. S. Li Y. T. Zhao X. M. Zheng X. Y. . (2026). Three novel Carotenoid Cleaving Dioxygenases from Nicotiana tabacum and their contributions to promoting aroma compound biosynthesis. Food Biosci. 79, 108714. doi: 10.1016/j.fbio.2026.108714 38826717

[B50] Liu S. H. Zhao X. S. Yang Q. Yang C. Q. Pan X. S. Zhang J. H. . (2021). Cloning and functional identification of monoterpene synthase gene NtTPS2 in tobacco. Biotech. Bull. 37, 132–141. doi: 10.13560/j.cnki.biotech.bull.1985.2020-1492

[B51] Luo W. Wang K. Luo J. Liu Y. Tong J. Qi M. . (2023). Limonene anti-TMV activity and its mode of action. Pestic. Biochem. Physiol. 194, 105512. doi: 10.1016/j.pestbp.2023.105512 37532363

[B52] Ma H. Steede T. Dewey R. E. Lewis R. S. (2024). Engineering sclareol production on the leaf surface of Nicotiana tabacum. J. Agric. Food. Chem. 10, 1021. doi: 10.1021/acs.jafc.4c02442 38840459

[B53] Meng Y. Wang Y. Guo W. Lei K. Chen Z. Xu H. . (2024). Analysis of the relationship between color and natural pigments of tobacco leaves during curing. Sci. Rep. 14, 166. doi: 10.1038/s41598-023-50801-1 38167588 PMC10762081

[B54] Mischko W. Hirte M. Roehrer S. Engelhardt H. Mehlmer N. Minceva M. . (2018). Modular biomanufacturing for a sustainable production of terpenoid-based insect deterrents. Green Chem. 20, 2637–2650. doi: 10.1039/C8GC00434J

[B55] Nugroho L. H. Peltenburg-Looman A. M. G. Verberne M. C. Verpoorte R. (2002). Is accumulation of sesquiterpenoid phytoalexins induced in tobacco plants constitutively producing salicylic acid? Plant Sci. 162, 989–993. doi: 10.1016/S0168-9452(02)00049-3

[B56] Oliveira J. A. C. Fernandes L. A. Figueiredo K. G. Corrêa E. J. A. Lima L. H. F. Alves D. S. . (2024). Effects of essential oils on biological characteristics and potential molecular targets in Spodoptera frugiperda. Plants (Basel) 13, 1801. doi: 10.3390/plants13131801 38999641 PMC11244083

[B57] Popova V. Ivanova T. Prokopov T. Nikolova M. Stoyanova A. Zheljazkov V. D. (2019a). Carotenoid-related volatile compounds of tobacco (Nicotiana tabacum L.) essential oils. Molecules 24, 3446. doi: 10.3390/molecules24193446 31547525 PMC6804150

[B58] Popova V. Ivanova T. A. Stoyanova A. S. Nikolova V. V. Docheva M. H. Hristeva T. H. . (2020). Chemical constituents in leaves and aroma products of Nicotiana rustica L. tobacco. Int. J. Food Stud. 9, 146–159. doi: 10.7455/ijfs/9.1.2020.a2 42171391

[B59] Popova V. Ivanova T. Stoyanova A. Nikolova V. Hristeva T. Docheva M. . (2019b). Polyphenols and triterpenes in leaves and extracts from three Nicotiana species. J. App. Biol. Biotech. 7, 45–49. doi: 10.7324/JABB.2019.70508

[B60] Popova V. Ivanova T. Stoyanova A. Nikolova V. Hristeva T. Gochev V. . (2019c). Terpenoids in the essential oil and concentrated aromatic products obtained from Nicotiana glutinosa L. Leaves. Molecules 25, 30. doi: 10.3390/molecules25010030 31861797 PMC6983188

[B61] Rabara R. C. Kudithipudi C. Timko M. P. (2023). Identification of terpene-related biosynthetic gene clusters in tobacco through computational-based genomic, transcriptomic, and metabolic analyses. Agronomy 13, 1632. doi: 10.3390/agronomy13061632 30654563

[B62] Raguso R. A. Schlumpberger B. O. Kaczorowski R. L. Holtsford T. P. (2006). Phylogenetic fragrance patterns in Nicotiana sections Alatae and Suaveolentes. Phytochemistry 67, 1931–1942. doi: 10.1016/j.phytochem.2006.05.038 16843507

[B63] Ralston L. Kwon S. T. Schoenbeck M. Ralston J. Schenk D. J. Coates R. M. . (2001). Cloning, heterologous expression, and functional characterization of 5-epi-aristolochene-1,3-dihydroxylase from tobacco (Nicotiana tabacum). Arch. Biochem. Biophys. 393, 222–235. doi: 10.1006/abbi.2001.2483 11556809

[B64] Romsuk J. Srisawat P. Robertlee J. Yasumoto S. Miura K. Muranaka T. . (2024). Heterologous production of corosolic acid, a phyto-insulin, in agroinfiltrated Nicotiana benthamiana leaves. Plant Biotechnol. J. 41, 277–288. doi: 10.5511/plantbiotechnology.24.0420a 40115767 PMC11921146

[B65] Roncero A. M. Tobal I. E. Moro R. F. Díez D. Marcos I. S. (2018). Halimane diterpenoids: sources, structures, nomenclature and biological activities. Nat. Prod. Rep. 35, 955–991. doi: 10.1039/c8np00016f 29701206

[B66] Sallaud C. Giacalone C. Töpfer R. Goepfert S. Bakaher N. Rösti S. . (2012). Characterization of two genes for the biosynthesis of the labdane diterpene Z-abienol in tobacco (Nicotiana tabacum) glandular trichomes. Plant J. 72, 1–17. doi: 10.1111/j.1365-313X.2012.05068.x 22672125

[B67] Seo S. Gomi K. Kaku H. Abe H. Seto H. Nakatsu S. . (2012). Identification of natural diterpenes that inhibit bacterial wilt disease in tobacco, tomato and Arabidopsis. Plant Cell Physiol. 53, 1432–1444. doi: 10.1093/pcp/pcs085 22685082

[B68] Sha Y. Yu S. Cai Z. Zhang Z. Song K. K. Yang L. (2026). Genome-wide analysis and characterization of CYP450 gene family in tobacco (Nicotiana tabacum L). Plant Mol. Biol. 44, 52. doi: 10.1007/s11105-025-01664-5 30311153

[B69] Shan Z. S. Wei Z. Jian G. T. Jian X. P. Dong L. Z. Liu Y. . (2016). 14-Noreudesmanesesquiterpenes from leaves of Nicotiana tabacum and their antiviral activity. Phytochem. Lett. 17, 173–176. doi: 10.1016/j.phytol.2016.07.019

[B70] Shang S. Shi J. Tang J. Jiang J. Zhao W. Zheng X. . (2019). New isolates from leaves of Nicotiana tabacum and their biological activities. Nat. Prod. Res. 33, 1577–1583. doi: 10.1080/14786419.2018.1425840 29350056

[B71] Shang S. Z. Zhao W. Tang J. G. Xu X. M. Sun H. D. Pu J. X. . (2016). Antiviral sesquiterpenes from leaves of Nicotiana tabacum. Fitoterapia 108, 1–4. doi: 10.1016/j.fitote.2015.11.004 26581121

[B72] Shen Q. P. Xu X. M. Li L. Zhao W. Xiang N. J. Yang G. Y. . (2016a). Sesquiterpenes from the leaves of Nicotiana tabacum and their anti-tobacco mosaic virus activity. Chin. Chem. Lett. 27, 753–756. doi: 10.1016/j.cclet.2016.01.048 26727192

[B73] Shen Q. P. Xu X. Liu C. Zhao W. Xiang N. Chen Y. . (2016b). Two new sesquiterpenes from the leaves of Nicotiana tabacum and their anti-tobacco mosaic virus activities. Nat. Prod. Res. 30, 2545–2550. doi: 10.1080/14786419.2015.1120729 26727192

[B74] Simpson R. D. Johnson A. W. Severson R. F. (1985). Leaf surface chemistry of tobacco budworm resistant and susceptible tobacco grown in the field and greenhouse at different fertilization rates. Tob. Sci. 29, 44–46. Available online at: https://www.coresta.org/abstracts/leaf-surface-chemistry-tobacco-budworm-resistant-and-susceptible-tobacco-grown-field-and-0.

[B75] Sommer S. Lang L. M. Drummond L. Buchhaupt M. Fraatz M. A. Zorn H. (2022). Odor characteristics of novel non-canonical terpenes. Molecules 27, 3827. doi: 10.3390/molecules27123827 35744956 PMC9230113

[B76] Song S. Gao Y. Fan P. Lin F. Zhou H. Li Z. . (2025). Mutations in active surface sites of NtGGPPS1 enhance carotenoid biosynthesis and drought resistance in Nicotiana tabacum. BMC Plant Biol. 25, 1296. doi: 10.1186/s12870-025-07337-5 41039249 PMC12492537

[B77] Starks C. M. Back K. Chappell J. Noel J. P. (1997). Structural basis for cyclic terpene biosynthesis by tobacco 5-epi-aristolochene synthase. Science 277, 1815–1820. doi: 10.1126/science.277.5333.1815 9295271

[B78] Ting H. M. Delatte T. L. Kolkman P. Misas-Villamil J. C. van der Hoorn R. A. Bouwmeester H. J. . (2015). SNARE-RNAi results in higher terpene emission from ectopically expressed caryophyllene synthase in Nicotiana benthamiana. Mol. Plant 8, 454–466. doi: 10.1016/j.molp.2015.01.006 25598143

[B79] Vranová E. Coman D. Gruissem W. (2013). Network analysis of the MVA and MEP pathways for isoprenoid synthesis. Annu. Rev. Plant Biol. 64, 665–700. doi: 10.1146/annurev-arplant-050312-120116 23451776

[B80] Wahlberg I. Enzell C. R. (1987). Tobacco isoprenoids. Nat. Prod. Rep. 4, 237–276. doi: 10.1039/np9870400237 3313125

[B81] Wang B. Dong W. Li H. D'Onofrio C. Bai P. Chen R. . (2022). Molecular basis of (E)-β-farnesene-mediated aphid location in the predator Eupeodes corollae. Curr. Biol. 32, 951–962. doi: 10.1016/j.cub.2021.12.054 35065682

[B82] Wang E. Wagner G. J. (2003). Elucidation of the functions of genes central to diterpene metabolism in tobacco trichomes using posttranscriptional gene silencing. Planta 216, 686–691. doi: 10.1007/s00425-002-0904-4 12569411

[B83] Wang E. Wang R. DeParasis J. Loughrin J. H. Gan S. Wagner G. J. (2001). Suppression of a P450 hydroxylase gene in plant trichome glands enhances natural-product-based aphid resistance. Nat. Biotechnol. 19, 371–374. doi: 10.1038/86770 11283597

[B84] Wang G. P. Yu X. D. Fan J. Wang C. S. Xia L. Q. (2015). Expressing an (E)-β-farnesene synthase in the chloroplast of tobacco affects the preference of green peach aphid and its parasitoid. Integr. Plant Biol. 57, 770–782. doi: 10.1111/jipb.12319 25644472

[B85] Wang Z. Zhang R. Yang Q. Zhang J. Zhao Y. Zheng Y. . (2021). Recent advances in the biosynthesis of isoprenoids in engineered Saccharomyces cerevisiae. Adv. Appl. Microbiol. 114, 1–35. doi: 10.1016/bs.aambs.2020.11.001 33934850

[B86] Wu J. Bu M. Zong Y. Tu Z. Cheng Y. Li H. (2024). Overexpression of the Liriodendron tulipifera TPS32 gene in tobacco enhances terpenoid compounds synthesis. Front. Plant Sci. 15. doi: 10.3389/fpls.2024.1445103 39354939 PMC11442295

[B87] Wu S. Liu F. Zeng W. Xiao Z. Li J. Teng K. . (2022). Evaluation of floral-derived volatile blend for attracting aphid parasitoids and lady beetles in the tobacco fields. Biol. Control 172, 104979. doi: 10.1016/j.biocontrol.2022.104979 38826717

[B88] Wu X. Singh S. K. Patra B. Wang J. Pattanaik S. Yuan L. (2025). An overview of the regulation of specialized metabolism in tobacco. Curr. Plant Biol. 41, 100431. doi: 10.1016/j.cpb.2024.100431 38826717

[B89] Xiao Q. Zhu Y. Cui G. Zhang X. Hu R. Deng Z. . (2022). A comparative study of flavonoids and carotenoids revealed metabolite responses for various flower colorations between Nicotiana tabacum L. and Nicotiana rustica L. Front. Plant Sci. 13. doi: 10.3389/fpls.2022.828042 35548319 PMC9083207

[B90] Xu M. Du Y. Hou X. Zhang Z. Yan N. (2024a). Chemical structures, biosynthesis, bioactivities, and utilisation values for the diterpenes produced in tobacco trichomes. Phytochemistry 223, 114117. doi: 10.1016/j.phytochem.2024.114117 38697243

[B91] Xu B. Huang J.-P. Peng G. Cao W. Liu Z. Chen Y. . (2024c). Total biosynthesis of the medicinal triterpenoid saponin astragalosides. Nat. Plants 10, 1826–1837. doi: 10.1038/s41477-024-01827-4 39433972

[B92] Xu K. Wei X. Liu J. Wang J. Du Y. Ni L. (2022). Sesquiterpenoids and diterpenoids from the flowers of Nicotiana tabacum L. and their antifungal activity. Rec. Nat. Prod. 16, 483–487. doi: 10.25135/rnp.293.2109.2211

[B93] Xu L. Xu Y. Jiang J. R. Cheng C. X. Yang W. W. Deng L. L. . (2024b). A novel AP2/ERF transcription factor, NtERF10, positively regulates plant height in tobacco. Transgenic Res. 33, 195–210. doi: 10.1007/s11248-024-00383-z 39105946 PMC11319389

[B94] Yan N. Du Y. Liu X. Zhang H. Liu Y. Zhang Z. (2019). A review on bioactivities of tobacco cembranoid diterpenes. Biomolecules 9, 30. doi: 10.3390/biom9010030 30654586 PMC6359560

[B95] Yan R. C. Jiang L. Hu M. Shen M. X. Liu M. Z. Shi Y. T. . (2025). Nicotabacins A–P, new cembrane-type diterpenes isolated from Nicotiana tabacum L. and their antifungal activities. J. Agric. Food. Chem. 73, 15637–15649. doi: 10.1021/acs.jafc.5c00994 40494642

[B96] Yan N. Liu Y. Gong D. Du Y. Zhang H. Zhang Z. (2015). Solanesol: a review of its resources, derivatives, bioactivities, medicinal applications, and biosynthesis. Phytochem. Rev. 14, 403–417. doi: 10.1007/s11101-015-9393-5 30311153

[B97] Yan N. Liu Y. Zhang H. Du Y. Liu X. Zhang Z. (2017). Solanesol biosynthesis in plants. Molecules 22, 510. doi: 10.3390/molecules22040510 28333111 PMC6154334

[B98] Yang C. Y. Geng C. A. Huang X. Y. Wang H. Xu H. B. Liang W. J. . (2014). Noreudesmane sesquiterpenoids from the leaves of Nicotiana tabacum. Fitoterapia 96, 81–87. doi: 10.1016/j.fitote.2014.04.010 24769287

[B99] Yang C. Halitschke R. O'Connor S. E. (2023). OXIDOSQUALENE CYCLASE 1 and 2 influence triterpene biosynthesis and defense in Nicotiana attenuata. Plant Physiol. 194, 2580–2599. doi: 10.1093/plphys/kiad643 38101922 PMC10980520

[B100] Yang C. Halitschke R. O'Connor S. E. Baldwin I. T. (2024a). Roles of three cytochrome P450 monooxygenases in triterpene biosynthesis and their potential impact on growth and development. Plant Physiol. 196, 1407–1425. doi: 10.1093/plphys/kiae399 39052981 PMC11444297

[B101] Yang Q. Liu S. H. Yang C. Q. Zeng R. M. Zhang Z. Liu D. Y. . (2022). Cloning and functional characterization of sesquiterpene ssynthase gene NtTPS21 in tobacco. Chin. Tob. Sci. 43, 39–46. 10.13496/j.issn.1007-5119.2022.03.007.

[B102] Yang P. S. Tang S. Y. Liu C. B. Ye L. Zhang F. M. He P. . (2019). Three new sesquiterpenes from the stems of Nicotiana tabacum and their bioactivities. Asian Nat. Prod. Res. 21, 109–116. doi: 10.1080/10286020.2017.1408597 29188722

[B103] Yang C. Q. Wu X. M. Yang A. G. Jiang X. Zhang Z. X. Li C. M. . (2024b). A tobacco sesquiterpene synthase NtTPS126 and its applications. Available online at: https://www.patentguru.com/cn/CN117363602A (Accessed March 25, 2026).

[B104] Yang C. Xie S. N. Ni L. Du Y. M. Liu S. Li M. Y. . (2021). Chemical constituents from Nicotiana tabacum L. and their antifungal activity. Nat. Prod. Commun. 16, 1–5. doi: 10.1177/1934578X211059578

[B105] Yang G. Y. Zhao W. Chen Y. K. Chen Z. Y. Hu Q. Miao M. M. (2013). Sesquiterpene glucosides from Nicotiana tabacum and their biological activity. Asian J. Chem. 25, 4932–4934. doi: 10.14233/ajchem.2013.14147

[B106] Yin J. L. Wong W. S. (2019). Production of santalenes and bergamotene in Nicotiana tabacum plants. PLoS One 14, e0203249. doi: 10.1371/journal.pone.0203249 30608920 PMC6319812

[B107] Yin J. L. Wong W. S. Jang I. C. Chua N. H. (2017). Co-expression of peppermint geranyl diphosphate synthase small subunit enhances monoterpene production in transgenic tobacco plants. New Phytol. 213, 1133–1144. doi: 10.1111/nph.14280 28079933

[B108] Zhai N. Wei X. Zheng Q. Zhang H. Xu Y. Xu G. . (2025). Whirly transcription factor NtWHY1 positively regulates the biosynthesis of cembranoid diterpenoids by directly targeting NtCBTS in tobacco. Physiol. Plant 177, e70280. doi: 10.1111/ppl.70280 40401685

[B109] Zhang T. Guo Y. Shi X. Yang Y. Chen J. Zhang Q. . (2020). Overexpression of LiTPS2 from a cultivar of lily (Lilium ‘Siberia’) enhances the monoterpenoids content in tobacco flowers. Plant Physiol. Biochem. 151, 391–399. doi: 10.1016/j.plaphy.2020.03.048 32278293

[B110] Zhang W. J. Pan X. Fu J. Cheng W. Lin H. Zhang W. J. . (2024). Phytochemicals derived from Nicotiana tabacum L. plant contribute to pharmaceutical development. Front. Pharmacol. 15, 1372456. doi: 10.3389/fphar.2024.1372456 38681197 PMC11045950

[B111] Zhang C. X. Wei F. Zhang G. H. Kong G. H. Cai X. H. (2025). Insight into the characteristic components of cigar tobacco. J. Agric. Food. Chem. 73, 2364–2372. doi: 10.1021/acs.jafc.4c12094 39818779

[B112] Zhang H. Zhang S. Yang Y. Jia H. Cui H. (2018). Metabolic flux engineering of cembratrien-ol production in both the glandular trichome and leaf mesophyll in Nicotiana tabacum. Plant Cell Physiol. 59, 566–574. doi: 10.1093/pcp/pcy004 29346685

[B113] Zhou W. Kügler A. McGale E. Haverkamp A. Knaden M. Guo H. . (2017). Tissue-specific emission of (E)-α-bergamotene helps resolve the dilemma when pollinators are also herbivores. Curr. Biol. 27, 1336–1341. doi: 10.1016/j.cub.2017.03.017 28434859

[B114] Zong Z. Q. Cao S. T. Wu X. Z. Liu Z. G. Zhang N. Chen X. Z. . (2023). Enhancing tobacco resistance to abiotic stress by exogenous substances: research progress. Chin. Agric. Sci. Bull. 39, 9. doi: 10.11924/j.issn.1000-6850.casb2022-0321

